# Modifying Rap1-signalling by targeting Pde6δ is neuroprotective in models of Alzheimer’s disease

**DOI:** 10.1186/s13024-018-0283-3

**Published:** 2018-09-26

**Authors:** Michael Dumbacher, Tom Van Dooren, Katrien Princen, Koen De Witte, Mélissa Farinelli, Sam Lievens, Jan Tavernier, Wim Dehaen, Stefaan Wera, Joris Winderickx, Sara Allasia, Amuri Kilonda, Stéphane Spieser, Arnaud Marchand, Patrick Chaltin, Casper C. Hoogenraad, Gerard Griffioen

**Affiliations:** 1 reMYND NV, Gaston Geenslaan 1, Leuven-Heverlee, 3001 Belgium; 2 E-Phy-Science, IPMC, 660 route des Lucioles, 06560 Sophia Antipolis, France; 3 Orionis Biosciences, Technologiepark 12B, Zwijnaarde-Ghent, 9052 Belgium; 4 Cytokine Receptor Lab, Flanders Institute of Biotechnology, Medical Biotechnology Center, Faculty of Medicine and Health Sciences, Ghent University, Albert Baertsoenkaai 3, 9000 Ghent, Belgium; 5 Department of Chemistry, KU Leuven, Celestijnenlaan 200f - box 2404, Leuven-Heverlee, 3001 Belgium; 6 ViroVet NV, Ambachtenlaan 1, Leuven-Heverlee, 3001 Belgium; 7 Department of Biology, Functional Biology, KU Leuven, Kasteelpark Arenberg 31 box 2433, Leuven-Heverlee, 3001 Belgium; 8 Cistim Leuven vzw, Gaston Geenslaan 2, Leuven-Heverlee, 3001 Belgium; 9 Center for Drug Design and Development (CD3), KU Leuven, Waaistraat 6, 3000 Leuven, Belgium; 10 Cell Biology, Department of Biology, Faculty of Science, Utrecht University, 3584CH Utrecht, The Netherlands

**Keywords:** Alzheimer’s disease, Hyperexcitability, Neuroprotection, Pde6δ, Rap1

## Abstract

**Background:**

Neuronal Ca^2+^ dyshomeostasis and hyperactivity play a central role in Alzheimer’s disease pathology and progression. Amyloid-beta together with non-genetic risk-factors of Alzheimer’s disease contributes to increased Ca^2+^ influx and aberrant neuronal activity, which accelerates neurodegeneration in a feed-forward fashion. As such, identifying new targets and drugs to modulate excessive Ca^2+^ signalling and neuronal hyperactivity, without overly suppressing them, has promising therapeutic potential.

**Methods:**

Here we show, using biochemical, electrophysiological, imaging, and behavioural tools, that pharmacological modulation of Rap1 signalling by inhibiting its interaction with Pde6δ normalises disease associated Ca^2+^ aberrations and neuronal activity, conferring neuroprotection in models of Alzheimer’s disease.

**Results:**

The newly identified inhibitors of the Rap1-Pde6δ interaction counteract AD phenotypes, by reconfiguring Rap1 signalling underlying synaptic efficacy, Ca^2+^ influx, and neuronal repolarisation, without adverse effects *in-cellulo* or in-vivo. Thus, modulation of Rap1 by Pde6δ accommodates key mechanisms underlying neuronal activity, and therefore represents a promising new drug target for early or late intervention in neurodegenerative disorders.

**Conclusion:**

Targeting the Pde6δ-Rap1 interaction has promising therapeutic potential for disorders characterised by neuronal hyperactivity, such as Alzheimer’s disease.

**Electronic supplementary material:**

The online version of this article (10.1186/s13024-018-0283-3) contains supplementary material, which is available to authorized users.

## Background

Neuronal hyperactivity and chronically elevated cytosolic calcium [Ca^2+^]_i_ are among the earliest pathological events in both familial and sporadic Alzheimer’s disease (AD) [1–4]. Amyloid-beta (Aβ), a peptide which progressively aggregates around neurons during the disease, appears to play an important role in driving these aberrations, as in the vicinity of Aβ accumulations (or plaques), where concentrations of soluble oligomeric Aβ (Aβo) are highest, neurons have a tendency to be hyperactive [5]. These AD associated changes in neuronal activity appear to involve, at least in part, a direct or indirect modulation of Ca^2+^ permeable receptors and ion channels as well as the disruption of lipid barriers by Aβo [6].

Apart from Aβ, also Tau, the second major aggregating protein crucial for AD progression, has signalling functions, involving Ca^2+^, and plays a role in excitability and network synchronisation [7, 8]. Of particular interest in this regard is dendritic Tau, which facilitates Ca^2+^ influx during Aβ induced neurotoxicity [7, 9]. An additional indication for the prominent role of Tau in AD is that, in animal models of the disease, the depletion of the former protein prevented Aβ associated neuronal hyperactivity and cognitive deficits [10, 11]. Thus, the interplay between Aβ and Tau in AD is essential for driving Ca^2+^ aberrations and excessive neuronal activity.

Moreover, neuronal hyperactivity and Ca^2+^ dyshomeostasis are not only the consequences of pathological Aβ and Tau but in turn also accelerate their formation. Aberrant Ca^2+^ signalling was shown to facilitate Tau phosphorylation [12] and Aβo production [13, 14], the precursors of the well-known AD hallmarks – neurofibrillary tangles and amyloid plaques respectively. Hence, it can be envisaged that risk factors for sporadic AD, which impair Ca^2+^ homeostasis (most notably ageing) [1, 6], set-off a neurotoxic cascade leading to neuronal hyperexcitability and aberrant network activity resulting in enhanced formation of Aβo and Tau phosphorylation. These aberrations in turn reinforce the disease cascade in a feed-forward fashion, bringing about progressive neuronal degeneration [15]. In case of familial AD, the same pathological sequence of events can be directly and potently initiated by genetic mutations which increase Aβo formation [16, 17].

The importance of excessive neuronal activity in light of the pathology is emphasised by recent findings linking enhanced cognitive deficits, accelerated symptoms and greater neuronal loss in AD to patients suffering from spontaneous non-motoric seizures (10–22% of AD population), compared to other AD patients [18]. In addition, treatments targeting abnormal neuronal activity, including the anti-epileptic drug levetiracetam, have shown to improve cognitive performance in mouse models of the disease [10, 19] and are currently under investigation in patients with AD during clinical trials (e.g.: NCT02002819) [20].

Collectively, these mechanistic underpinnings indicate that normalising Ca^2+^ homeostasis and hyperactivity represent a promising therapeutic paradigm. However, given the fundamental nature of Ca^2+^ signalling in cell physiology, targeting such deregulations in a safe fashion represents a major challenge. Here we addressed this problem by screening directly for therapeutic compounds using a neuronal cell based phenotypic screen that mimics crucial mechanisms underlying AD and by subsequently identifying their corresponding cellular target conferring neuroprotection.

## Methods

### Mice, drug treatment, behavioural testing and termination

Human mutant transgenic APP-V717I [21] (hAPP) and APP-V717I*PS1-A246E [22] (hAPP*PS1) mice in FVB/NxC57Bl/6 J background and age-, sex- and background-matched wild-type (WT) mice with ad libitum access to food and water were used. Breeding and housing for electrophysiological studies was in conventional 12 h light/dark cycle. Housing of mice for behaviour and biochemistry was in inverted 12 h light/dark cycle. All animals used in this study were female and four mice were housed per cage. Overexpression of human transgenes is steered by the mouse neuron-specific Thy1 gene promotor. All experimental procedures were performed in accordance with the Guide for the Care and Use of Laboratory Animals (NRC, 2011) and the European Communities Council Directive of September 22nd, 2010. REM0043039 was dissolved in liposomes (Phares, Switzerland).

For chronic treatment of REM, hAPP and hAPP*PS1 mice were injected daily subcutaneously (sc) with vehicle or compound at 20 mg/kg. For acute REM effects, mice were dosed orally at 30 mg/kg achieving (similarly to sc dosing) a sufficiently high free compound concentration in brain. Pharmacokinetic assessments, prior to the commencement of studies, had indicated that both dosing regimens resulted in sufficient compound levels for target engagement (data not shown).

hAPP*PS1 used for biochemistry were dosed during 14 consecutive days before termination at the age of 6 months. For behaviour studies, daily treatment of hAPP and control mice started at the age of 4.5 months. After 8 weeks treatment (around 8 months of age) hAPP and control mice were tested in the Morris water maze (MWM) paradigm. Therefore, mice were trained to find the hidden escape platform (10 cm diameter, 1.5 cm submerged below the surface) in a pool (160 cm diameter) filled with white opaque water (23–24 °C) during 4 consecutive days and 4 trials per day (with a maximum of 90 s trial and an inter-trial interval of 120 min). Both intra- and extra-maze cues were present. During the first day mice were allowed to sit on the platform for 10 s before being taken out of the pool. Mice that did not mount the platform were gently guided to it. The platform location remained constant throughout the 4 training days. However, the drop location of the mice varied semi-randomly among trials. For the probe trial on day 5 the platform was removed, and mice were allowed to swim for 60 s. The swimming pattern of the mice was video-monitored using a CCD camera and analysed using dedicated software (EthoVision, Noldus, The Netherlands). Search path (cm) and the annulus crossing index (ACI, defined as the frequency of crossing the target platform region minus the averaged imaginary platform regions in the other quadrants) were used as primary read-out during learning and probe trial, respectively. At the end of the study, all mice were terminated.

### Cell culture

For construction of hTAU-P301L cells a TAU expression plasmid was constructed by sub-cloning the cDNA of human TAU-P301L (encoding for TAU with proline 301 substituted by a leucine residue) into mammalian expression vector pcDNA3.1 resulting in plasmid pcDNA3.1-TAU P301L. Plasmid pcDNA3.1-TAU-P301L were transfected into human BE(2)-M17neuroblastoma cells (ATCC No. CRL-2267) with the plasmids stably integrated into the genome were selected. These resulted in the cell line referred to as hTau-P301L. Expression of the hTAU-P301L gene in the cell lines was confirmed by Western blot analysis (Additional file 1: Figure S1a).

hTAU-P301L cells were cultured in culture medium containing Opti-MEM Reduced Serum Medium with phenol red supplemented with 1 mM sodium pyruvate, 1 x non-essential amino acids, 500 μg/ml G418 0,5 x antibiotic/antimycotic, antibiotics and 10% foetal calf serum (FCS).

For the cellular toxicity assay hTAU-P301L cells were seeded at 2500 cells/cm^2^ in 96-well microplates, in experiment medium containing Opti-MEM Reduced Serum Medium without phenol red supplemented with 1 mM sodium pyruvate, 1 x non-essential amino acids, 500 μg/ml G418 0,5 x antibiotic/antimycotic, antibiotics and 1% FCS. After 3 h of incubation at 37 °C/5% CO_2_, 100 μl of experiment medium without FCS supplemented with all-trans retinoic acid (ATRA) (final concentration 3.75 μM) and compound dissolved in dimethyl sulfoxide (DMSO) or vehicle alone (final concentration DMSO 2%) was added. The cells were further incubated for 7 days at 37 °C/5% CO_2_. Subsequently, Lactate dehydrogenase (LDH) activity was determined using Promega Cytotox 96 Non-Radioactive cytotoxicity assay (Cat. G1780), according the supplier’s instructions and the percentage LDH in the growth medium was calculated as a measure for toxicity using the following formula (slopes are calculated from the measured kinetic A492 values in function of time):

$$ Toxicity=\frac{slope\ of\ released\  LDH\ \left( dead\ cells\right)}{slope\ of\ total\  LDH\ \left( dead+ viable\ cells\right)}. $$

Primary hippocampal cultures were prepared from CD-1 (Swiss) embryonic day 16 (E16) mouse brains and plated on culture plates or glass coverslips coated with (0.1 mg/ml) poly-D-lysine (PDL) and (0.002 mg/ml) laminin or PDL pre-coated plates (Greiner) were used. Cultures were grown in serum free Neurobasal medium (NB, Gibco) supplemented with B27 (Gibco), glutamax (Gibco) and penicillin/streptomycin (50 u/ml) in multi-well plates at a density of 0.15 × 10^6^ cells per ml. Culture medium was refreshed twice a week.

### siRNA transfection

For gene silencing experiments hTAU-P301L neuroblastoma cells were plated out 24 h before transfection in 6-well plates. Cells were with Lipofect-amine® RNAiMAX (Thermo Fisher) according to the Thermo Fisher’s “forward transfection protocol” with a final siRNA concentration of 25 pmol. RNA sequences used were siRNA Rap1A (Thermo Fisher, #4390771, s11779), siRNA retinal rod rhodopsin-sensitive cGMP 3′,5′-cyclic phosphodiesterase subunit delta (Pde6δ, protein ID: O43924) (Thermo Fisher, #4390771, s10207) and scrambled negative siRNA control (Thermo Fisher, #4390843). Twenty-four hours post-transfection the cells were re-plated into 96-well microplates for the toxicity assay.

### Biochemical pull-down: Pde6δ and REM-MTX interaction assay

The cDNA encoding PDE6δ was sub-cloned into the vector pPR-IBA102 in frame with the upstream Strep tag. The resulting construct was used to express protein using a Mini Expressway cell-free *E. coli* expression system (Life Technologies) according to the manufacturer’s instructions. Correct expression was confirmed using Western blot analysis with the StrepMAB-Classic antibody (data not shown).

For the interaction assay, *E. coli* cell-free lysate containing Strep-PDE6δ and methotrexate fused REM compound (REM-MTX) or the corresponding amount of DMF were mixed and incubated on ice for 3 h to achieve binding of the compound to the target. Subsequently, equilibrated Strep-Tactin Sepharose beads were added and incubated for 90 min at 4 °C with over-end rotation. Excess compound was removed by washing the beads 3 times with wash buffer (20 mM Tris-Cl pH 7.4, 150 mM NaCl). After removal of the last wash *E. coli* cell-free lysate containing His-DHFR in wash buffer was added to each sample, followed by incubation for 60 min at 4 °C with over-end rotation. After flow through removal and 3 subsequent washes, the beads were eluted with elution buffer containing 10 mM Desthiobiotin for 30 min on ice. Samples were mixed with SDS sample buffer for SDS-PAGE analysis and Western blot and analysed with an Anti-HisG antibody.

### Affinity purification coupled with mass spectrometry to identify interactors with Pde6δ

The cDNA encoding human Pde6δ was fused with a Strep-HA tag encoding DNA sequence and sub-cloned into a standard expression vector. The bait sequence was verified by sequencing. For constitutive overexpression, the expression vector containing the gene encoding the bait Pde6δ-Strep-HA was transfected into HEK293 cells and selected for stable integration. Bait expression and solubility was monitored by immunoblotting using anti-HA antibodies. For affinity purification, protein extracts were prepared from control (HEK293 cells not expressing Pde6δ-Strep-HA), vehicle (DMSO) and compound treated Pde6δ-Strep-HA bearing cells under specific lysis conditions and the fusion protein was pulled-down using Strep-Tactin Sepharose beads. Affinity purification and mass spectrometry analysis of Pde6δ-Strep-HA expressed in HEK293 cells was performed with three biological replicates. For label free quantitative mass spectrometry analysis samples were analysed on a Thermo LTQ Orbitrap XL spectrometer using a C18 column, ESI and a 60 min gradient. The signals’ intensity is provided as 2 Log values and correlates to the concentration of the detected mass (number/amount of peptide). The variation within the biological replicates for control, vehicle-treated Pde6δ pulldown, and REM treated Pde6δ pulldown was below 10%. A Co-precipitation threshold of 2 (Pde6δ vehicle /control value) is set to decide on the interacting properties of a protein to Pde6δ. For interacting proteins, signals were background corrected and REM treated samples were normalised to the DMSO control samples to identify for Pde6δ-protein interactions inhibited by REM.

### Ca^2+^ recordings

For basal Ca^2+^ measurement in the neuroblastoma toxicity model, cells were loaded with Fura-2 AM (Sigma-Aldrich), a cell permeable fluorescent probe for Ca^2+^ at day 6 of the experiment. Briefly, Fura-2-AM was dissolved in DMSO plus 20% pluronic acid (0.025% final assay concentration (Invitrogen)) in a 1:1 ratio and diluted in medium to a final concentration of 0.5 μM. Probenecid (Sigma-Aldrich) is added to this loading medium at a final concentration of 2.5 μM. Then, culture medium is replaced by loading medium and after incubation for 1 h at 37 °C cells in the dark were washed and incubated in HBSS (Gibco) supplemented with 0.2% FCS (Gibco) and 0.02 M HEPES (Gibco) before Ca^2+^ being recorded in the FlexStation 3 (Molecular Devices). Cells were excited at 340 nm (Ca^2+^ bound Fura-2) and 380 nm (Ca^2+^ unbound Fura-2) of the FlexStation 3 with emission at 510 nm (cut-off filter 475 nm) in well scan modus (9 points/well).

Days in-vitro (DIV) > 14 hippocampal neurons were treated 24 h prior to experiment with 1.5 μM REM or equivalent amount of vehicle and were present throughout the experiment. One hour prior to Ca^2+^ recordings, primary neurons were incubated with loading medium containing 2 μM of the cell permeable Ca^2+^-indicator Fura-2 AM, 0.02% pluronic acid (F-127) (Invitrogen) and 2.5 μM Probenecid (Sigma-Aldrich) for 30 min at 37 °C in the dark. After loading, cells were washed and incubated in HBSS (Gibco) supplemented with 10 mM HEPES (Gibco) (with or without 12.5 μM nifedipine/vehicle) and/or 1.5 μM REM/vehicle for 30 min, in the dark, at room temperature. Cells were then excited at 340 nm and 380 nm in time with the flex mode of the FlexStation 3, recording 4 reads per well every 3.6 s. Emissions were collected at 510 nm (cut-off filter 475 nm). After 60 s of “background reading”, HBSS-HEPES containing high KCl (with adjusted monovalent ion concentration – variable amounts of NaCl/KCl, as previously described [23]) or HBSS-HEPES “control solution” was added to the cells and emissions were read further for four minutes.

Overall, changes in [Ca^2+^]_i_ were quantified, by making the ratio of changes in the amount of cytosolic Ca^2+^ bound Fura-2 (fluorescence intensity at 340 nm) relative to the amount of Ca^2+^ unbound Fura-2 (fluorescence intensity at 380 nm). Area under the curves (AUC) was calculated after normalising the data to the average baseline (first 60 s of recording) values. Data was processed with SoftMax Pro 5.4.6 software (Molecular Devices).

### Electrophysiology (acute brain slices, sAP, firing rate, LTD)

Acute sagittal brain slices from WT or hAPP mice were prepared by decapitation of the mice after isoflurane anaesthesia. Brains were removed quickly and immersed during 3–4 min in ice-cold freshly prepared cutting artificial cerebrospinal fluid (cutting aCSF) containing (in mM) 214 sucrose, 2.5 KCl, 2 CaCl_2_, 2 MgSO_4_, 1.25 NaH_2_PO_2_, 26 NaHCO_3_ and 10 glucose and oxygenated with 95% O_2_/5% CO_2_. Sagittal 350 μm slices were generated using a vibratome (VT 1000S; Leica Microsystems) and were incubated in standard carboxygenated aCSF (in mM: 125 NaCl, 2.5 KCl, 2 CaCl_2_, 2 MgSO_4_, 1.25 NaH_2_PO_2_, 26 NaHCO_3_ and 10 glucose, osmolarity 305 mOsm) at 34 °C for 20 min. The incubation continued at room temperature (RT) for another hour before each slice was transferred to a submerged recording chamber and perfused continuously with carboxygenated aCSF.

Somatic or dendritic (approximately 250 μm from the soma) current-clamp recordings were performed on slices from mice aged 3–4 months at RT (24 to 28 °C). The slices were continuously perfused with carboxygenated standard aCSF. Depending on the experiment, aCSF was supplemented with control or test article REM0043039 at 2 μM. For whole-cell recordings, patch pipettes were filled with a solution containing (in mM) 140 K-gluconate, 5 NaCl, 2 MgCl_2_, 10 HEPES, 0.5 EGTA, 2 MgATP, 0.4 NaGTP, osmolarity 305, pH adjusted to 7.25 with KOH. The soma or dendrite of large CA1 pyramidal neurons were identified and patch-clamped after visual approach of the recording pipette using a combination of infrared light and differential interference contrast (DIC) optics. Patch electrodes had a resistance of around 5 and 14 MΩ when filled for somatic and dendritic recording, respectively. Recordings were terminated when the series resistances exceeded 40 MΩ. Signals were digitised, and low-pass filtered at 10 kHz. The signal was amplified with an Axopatch 200B amplifier, digitised by a Digidata 1550 interface and sampled with Clampex 10 (Molecular Devices).

For the single AP experiment, a single spike was elicited by injecting a 2 milliseconds depolarising current pulse. The following action potential (AP) parameters were analysed: AP area, AP amplitude, AP delay, AP half-width, AP rising slope, AP decay slope, afterhyperpolarisation (AHP) amplitude (calculated by subtracting the minimum value following the AP from the resting membrane potential), AHP half-width and AHP time-to-peak. Somatic single AP recordings were performed at baseline and after 25 min vehicle or compound perfusion. Dendritic single AP recording occurred after at least one hour vehicle or compound incubation and throughout the experiment in perfusion.

Dendritic firing rate of CA1 cells was recorded in response to hyperpolarising and depolarising steps (− 0.2 to + 0.45 nA, steps of 0.05 nA) after at least one hour incubation with vehicle or compound at 2 μM and throughout the experiment in perfusion. The mean number of action potentials (firing rate) was plotted in function of current step intensity. In addition, AP amplitude, AP onset, AP threshold, AHP amplitude, input resistance and current-voltage relation were analysed. Results were corrected for vehicle effect.

Somatic voltage-clamp recording of CA1 cells was performed to determine total (P1) and I_A_-type potassium (K^+^) currents (P2). Electrodes were filled with an intracellular solution containing (in mM) 145 KCl, 1 MgCl_2_, 10 EGTA, 0.2 CaCl_2_, and 10 HEPES buffer (Sigma). Tetrodotoxin (TTX; 1 mM) and CdCl_2_ (0.3 mM) were added into the aCSF to block Na^+^ voltage dependent channels and Ca^2+^ channels, respectively. Membrane potential was held at − 60 mV. Depolarising potential steps were preceded by 300 milliseconds hyperpolarising pulse at − 120 mV (voltage protocol P1) to evoke outward currents including I_A_. I_A_ was inactivated by a 50 milliseconds prepulse at + 10 mV (voltage protocol P2). I_A_ was obtained by subtracting the current evoked from P2 from that evoked from P1 (Klee et al. 1997; Numann et al. 1987). The amplitude of I_A_ was measured at the peak of the current (at 20 milliseconds after the onset of testing pulses).

MGluR-mediated long-term depression (LTD) was induced by perfusion of 50 μM (S)-3,4-dihydroxyphenylglycine (DHPG; Tocris) to WT (C57Bl/6 J background, 6–8 weeks old) brain slices for 5 min. DHPG was dissolved in H_2_O at a stock concentration of 50 mM and aliquots were stored at − 80 °C until dilution into aCSF. Field excitatory postsynaptic potentials (fEPSPs) recordings were performed in the stratum radiatum using a borosilicate micropipette filled with aCSF in a submerged chamber continuously perfused with a CSF at 1.1 ml/minute. The signal was amplified with an Axopatch 200B amplifier, digitised by a Digidata 155 interface and sampled with Clampex 10 (Molecular Devices). Before baseline fEPSP recording and perfusion of DHPG, brain slices were preincubated in aCSF supplemented with vehicle or compound at 2 μM for 80 min. Perfusion of vehicle or compound continued during fEPSP recording up to 60 min after DHPG addition.

For the biochemical analysis of DHPG-induced LTD in primary cells, DIV 19–22 hippocampal neurons were pre-treated > 30 min prior to 50 μM DHPG with 1 μM TTX (Tocris) as well as 1.5 μM REM0043039 or vehicle (DMSO). DHPG was weighed freshly to make up a stock of 25 mM in sterile H_2_O. After 30 min, DHPG added to the cells at a final concentration of 50 μM for 10 min. Cells were lysed on ice for ten minutes in RIPA Buffer (Thermo Fisher) containing 1× Halt^TM^ Protease and Phosphatase Inhibitor Cocktail (Thermo Fisher) and debris was removed by centrifuging the homogenate at 14.000 x g for 10 min at 4 °C. Samples were stored at − 80 °C until further use.

### Tissue/cell processing and Western blot analysis

Cortex samples of hAPP*PS1 mice were homogenised using a potter-type mechanical homogeniser (VOS 14 S40, rate ~ 750 rpm VWR) in 6.5 weight-volumes of cold tris-protease-phosphatase-inhibition buffer (TPPI-buffer) containing (in mM) 50 Tris-HCl (pH 8.1), 250 saccharose, 3 MgCl_2_, 1 EDTA, 1 EGTA and a cocktail of Halt^TM^ protease and phosphatase inhibitors (Thermo Fisher). Whole extract was aliquoted and stored at − 80 °C until further use.

Tissue whole protein extracts were diluted with an equal volume of SDS-PAGE sample buffer (containing final concentrations of 1% (*w*/*v*) SDS and 2.5% (*v*/v) 2-mercaptoethanol) and were denatured and reduced by incubation at 95 °C for 10 min. The same was done for Cell extracts after adjusting amounts of protein and volume for each sample (determined using Biorad’s DC protein assay (Biorad)). Proteins were separated on 4–12% Bis-Tris or 7.5% Tris-HCl gels (Criterion XT Precast Gel, 26 well, 15 μl, 1.0 mm; Biorad). After semi-dry electrotransfer (iBlot™, Invitrogen) to PVDF-membranes (iBlot™ Gel Transfer Stacks, PVDF, Regular, Invitrogen), the membranes were incubated 2 h or overnight in tris-buffered saline (TBS, pH 7.6) with 0.1% (v/v) Tween-20 containing either 5% (*w*/*v*) non-fat dry milk or 5% bovine serum albumin (BSA) (depending on primary antibody). The next day, the blots were incubated with primary antibody for 2 h or overnight. After washing and incubation for at least 2 h with an HRP-conjugated secondary antibody (goat-anti-mouse or goat-anti-rabbit IgG, DAKO; goat-anti-rabbit IgG, Cell Signaling) blots were developed by the ECL detection system (LiteAblotR Plus ECL substrate, EuroClone; or SuperSignal West Femto Maximum Sensitivity Substrate, product 34096, Thermo Fisher) and images were recorded digitally (VisionWorks Acquisition, UVP) at different exposure times. Dedicated software (VisionWorks Analysis, UVP) was used for densitometric analysis.

Primary antibodies used: rabbit p44/42MAPK (ERK1/2) (Cell Signaling, #4695), rabbit phospho-p44/42MAPK (ERK1/2) (Thr202/Thr204) (Cell Signaling, #4370), rabbit alpha-Tubulin (Abcam, ab24246), rabbit GAPDH (Abcam), rabbit Rap1A (Novus Biologicals, NBP1–97-489), rabbit Pde6δ (Genetex, GTX109240), mouse AD2 (Biorad, #56484), pS262 Tau (ProSci, XBP-4276), pan-Tau (Tau5, Calbiochem, #577801), HT7 Tau (human Tau, Thermo Fisher, MN1000). HRP-conjugated secondary goat anti-rabbit (Dako), goat anti-mouse (Dako) and goat-anti-rabbit (Cell Signaling) antibodies were used.

### Docking procedures

All calculations were carried out using Schrodinger Suite software [24] with default parameters except when otherwise specified. High resolution X-ray crystal structure of farnesylated Rheb in complex with Pde6δ [25], PDB code 3T5G, was selected to perform molecular modelling calculations. Rheb protein atoms were deleted to give the starting structure of isolated Pde6δ in complex with farnesyl moiety fused to terminal Rheb cysteine residue. Protein preparation was then performed in order to add hydrogen atoms, to remove water molecules or to identify possible interacting water, to fix missing sidechain atoms or missing residues, to assess protonation state of ionisable amino acids. Docking simulations were performed using GlideXP (extra precision).

### Statistical analysis and data collection

To assess whether the means of two groups are statistically different from each other, we used an unpaired two-tailed t-test unless specified otherwise. Ordinary two-way ANOVA was applied to compare differences in the mean between groups with two independent variables followed by Sidak’s test for multiple comparisons as post-hoc analysis unless stated otherwise. Where appropriate, the Grubb’s method (alpha = 0.05) was used to identify outliers.

For all ex-vivo electrophysiology and in-vivo studies both animals and treatment were randomly allocated to a treatment group (digital randomisation occurred in Excel using the rand-function). In-vivo and ex-vivo practicalities and analysis of data was always performed in a blind manner after which the treatment groups were decoded by the study director.

All statistical analysis was done with the Prism 7 software (GraphPad). Values are given as mean ± standard error of the mean unless indicated otherwise. Error probabilities of *P* < 0.05 were considered statistically significant. Indication of *p*-value summaries: *p < 0.05, **p < 0.01, ***p < 0.001, ****p < 0.0001 or ####*p* < 0.0001.

### Target identification screen

A three-hybrid approach used to identify the target as described [26, 27]. To this end REM0043037 (B1) was fused with methotrexate, finally resulting in REM0044931 (B3), as a tool compound for identifying interacting proteins was prepared (see “Preparation of fusion compound used for the target identification screen”).

### Preparation of fusion compound used for the target identification screen

Abbreviations: CC – Column chromatography; DCM – Dichloromethane; TEA – Triethylamine; DMF – N,N-dimethylformamide; BOP: (benzotriazol-1-yloxy) tris (dimethylamino) -phosphonium hexafluorophosphate; TLC – Thin layer chromatography; DMSO. Analysis by LC-MS-UV was carried out on an Agilent system with a 3 × 150 mm Phenomenex Luna 5 μm column, flow 0.8 ml/minute; UV detection at 240 nm and 300 nm, neg. And pos. MS detection (150–1500 amu, fast mode); ambient temperature; gradient made from ACN and aq. 10 mM ammonium formate pH 9. MS (pos.), MS (neg.) and DAD information was recorded online. For MS spectra only the most abundant isotope signal is indicated; for UV spectra, only signals > 220 nm are indicated

### Preparation of compound 38

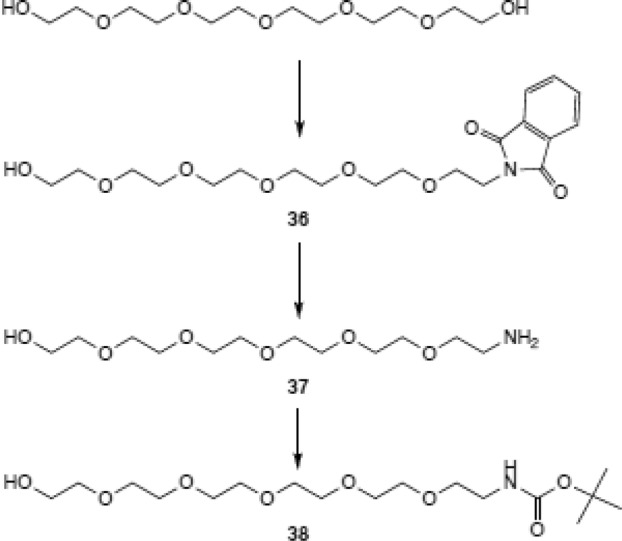



A solution of 28 g (0.1 mol) hexaethylene glycol, 27.5 g (0.105 mol) triphenylphosphine and 15.5 g (0.105 mol) phthalimide in 300 ml THF was treated with 20 g (0.1 mol) diisopropyl azodicarboxylate. The solution was stirred 45 min at 20 °C and filtered. The filtrate was evaporated and subjected to CC (350 g SiO_2_) with a DCM / MeOH gradient affording 18 g 36 as viscous, colorless oil. This compound was dissolved in 300 ml MeOH, treated with 15 ml hydrazine monohydrate and kept overnight at 50 °C. The suspension was filtered and evaporated: 11.5 g 37 as colorless oil. This oil was suspended in 75 ml CHCl3, filtered and evaporated. The residue was dissolved in 300 ml DCM and treated with 10 g (0.1 mol) TEA and 11 g (0.05 mol) di-tert-butyl dicarbonate. After 30 min at 20 °C, the solution was evaporated, and the residue was subjected to CC (200 g SiO_2_) with a DCM / MeOH gradient, yielding 15 g (40% overall) of 38 as a viscous, colorless oil.

### Preparation of compound 39 (= B2)

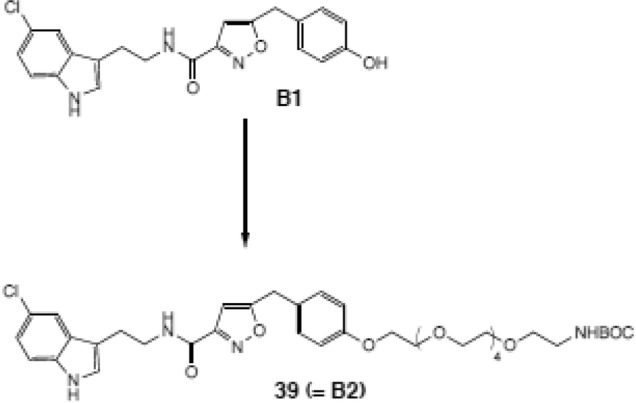



A suspension of 150 mg (0.375 mmol) compound B1 (synthesised by CISTIM), 150 mg (0.4 mmol) 38 and 190 mg (0.75 mmol) 1,1′-(azodicarbonyl)dipiperidine in 5 ml THF was heated at 50 °C and then treated with a solution of 150 mg (0.75 mmol) tributylphosphine in 1 ml THF. After 45 min at 50 °C, the suspension was filtered, and the filtrate was evaporated. The residue was subjected to CC (6 g SiO_2_) with a hexane / EtOAc gradient. The product-containing fractions were evaporated affording 250 mg of a white solid (containing about 30 wt % reduced 1,1′-(azodicarbonyl)dipiperidine). Fifty mg of this mixture were subjected to prep. TLC with EtOAc as eluent. The product-containing band was scratched off and eluted with EtOAc / MeOH 9:1 yielding 18 mg 39 as a colorless oil.

### Preparation of compound 40

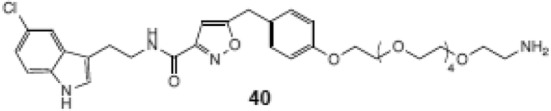



At 20 °C, 200 mg of the material obtained above (containing about 70% 39) were dissolved in 2.5 ml TFA / H_2_O 9:1. After 45 min, 10 ml toluene were added, and the mixture was evaporated. The residue was subjected to CC (6 g SiO_2_) with a DCM / MeOH gradient; after elution of by-products with 4% MeOH, 2% TEA was added to the eluent to elute the product. The product-containing fractions were evaporated, giving 125 mg 40 as colourless, viscous oil (63% yield based on B1).

### Preparation of compound 41

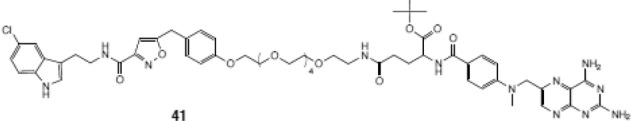



A solution of 125 mg (0.19 mmol) 40 and 105 mg (0.2 mmol) MTX (COOtBu) COOLi in 1.0 ml DMF was treated with 100 mg (0.22 mmol) BOP. After 30 min at RT, LC-MS indicated completion of the reaction. The reaction mixture was diluted with 8 ml MeOH/H_2_O 1:1 and purified by MPLC (250 ml MeOH / aq. 10 mM NH_3_ 1:3, 250 ml ACN / aq. 10 mM NH_3_ 4:6, 500 ml ACN / aq. 10 mM NH_3_ 1:1). The product containing fractions (analysed by LC-MS) were evaporated, co-evaporated with MeOH and then with DCM and dried: 170 mg (78%) product 41 as yellow, solid foam. LC (10–100% in 15 min): 8.2 min (96%) UV: 230 nm (max), 260 nm, 305 nm, 375 nm MS (pos): 1150 (M + 1) MS (neg): no signal detected.

### Preparation of fusion compound used for the target identification screen (alias 42)

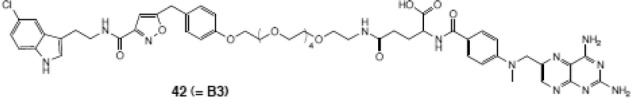



Solid 41 (170 mg, 0.145 mmol) was dissolved in 2.0 ml TFA/H_2_O 9:1 and stirred 50 min at 20 °C. The reaction mixture was cooled to 4 °C and neutralised by drop-wise addition of conc. aq. NH_3_ solution, diluted with 5 ml MeOH/H_2_O 1:1 and purified by MPLC (500 ml MeOH / aq. 10 mM NH_3_ 1:9, 500 ml ACN / aq. 10 mM NH_3_ 7:3). The product containing fractions were evaporated, co-evaporated with MeOH and dried: 120 mg (75%) of product 42 (REM0044931) as a yellow solid. ^1^H-NMR (300 MHz, CD_3_OD / CDCl_3_ 2:1): LC (10–100% in 15 min): 5.6 min (98%), UV: 230 nm (max), 260 nm, 305 nm, 375 nm, MS (neg.): 1092 (M-1), MS (pos): 1094 (M + 1).

## Results

### Identification of small molecule modifiers counteracting mechanisms underlying neurotoxicity in AD

**Fig. 1:**
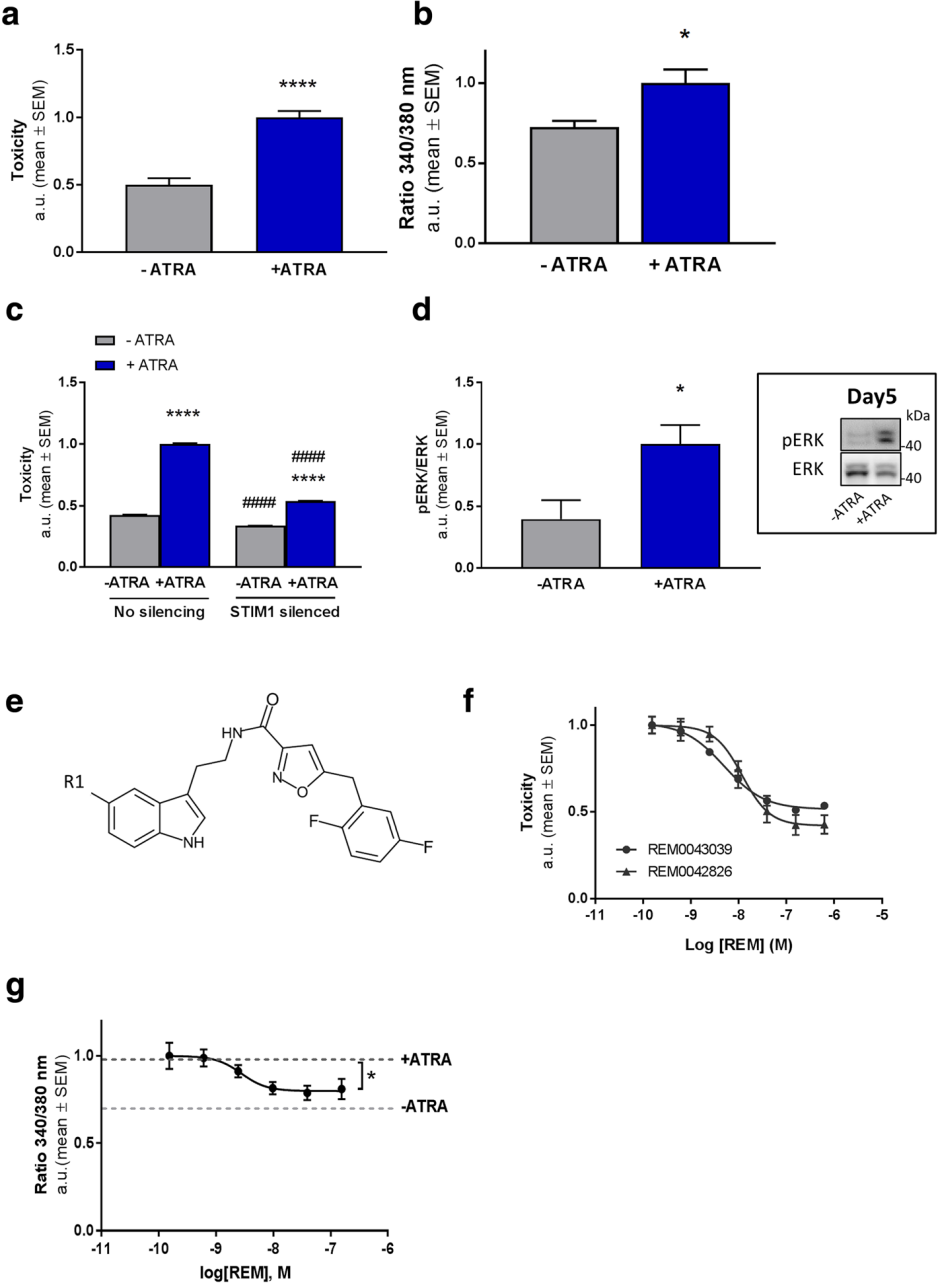
Development of an AD model for identifying neuroprotective compounds. **a** Toxicity was determined by quantifying LDH release in the medium in BE (2)-M17 neuroblastoma cells incubated for 7 days with or without ATRA. The ATRA induced toxicity in the cells represents the “toxicity assay” used in all following experiments (*n* = 5; +/− ATRA: *P* < 0.0001; *t* = 7,346 DF = 8). **b** Cytosolic Ca^2+^ levels quantified by Ca^2+^-indicator Fura-2 fluorescence ratio (340 nm/380 nm) in hTAU-P301L cells after 6 days with and without ATRA treatment in the toxicity assay (*n* = 3; *P* = 0.0435; *t* = 2.915; DF = 4). **c** Genetic silencing of STIM1 reduces toxicity in the toxicity assay. # denotes the effect of STIM1 silencing compared to unsilenced cells, * indicates the effect of ATRA treatment (silencing: *P* < 0.0001; DF = 1; F (1, 36)=4048; ATRA treatment: *P* < 0.0001; DF = 1; F (1, 36)=8006; Corrected for multiple comparison using Tukey’s test: +/−ATRA - silencing: *P* < 0.0001; q = 132.4; DF = 36; −ATRA +/− silencing: *P* < 0.0001; q = 20.68; DF = 36; +/−ATRA + silencing: P < 0.0001; q = 46.53; DF = 36; +ATRA +/− silencing: P < 0.0001; q = 106.6; DF = 36). **d** Increased MAPK signalling prior to toxicity measured by western blot in the cells treated with or without ATRA (day 5). Data are normalised to the “control” (−ATRA) condition (*n* = 4; *P* = 0.0322, *t* = 2.775, *DF* = 6). A representative immunoblot is shown in the inset next to the graph. **e** The chemical structure of the REM compounds. R1 could be either Cl (REM0042826) or F (REM0043039) (Additional file 2: Supplementary Methods). **f** Concentration-response curves of ATRA-toxicity reducing effect REM0042826 (EC_50_ = 12 nM) and REM0043039 (EC_50_ = 5 nM) in the toxicity assay (*n* = 7 or 5 for REM0043039 or REM0042826 respectively). **g** Concentration-response curve (*n* = 3) of the cytosolic Ca^2+^ reducing effect of REM, (EC_50_ of 3 nM) in the toxicity assay. The dotted grey line represents the average basal Ca^2+^ levels of vehicle treated cells in the model (One-way ANOVA, REM treatment: *P* = 0.0387; *F* = 3.386; *DF* = 5)

In order to identify new drug targets and therapeutics underlying AD we implemented a neuronal cell-based model of Ca^2+^-driven neurodegeneration. For this reason, we used neuroblastoma cells containing mutant human tau (TAU-P301L) as these cells were found to be more susceptible to toxic stimuli compared to control cells (Additional file 1: Figure S1a). Neurotoxicity in the model was induced by incubating the cells with ATRA [28]. Upon chronic exposure to ATRA a significant increase in toxicity within seven days in culture was consistently detected (Fig. 1a; Additional file 1: Figure S1b).

Quantification of Ca^2+^ indicator fura-2 fluorescence in the cells following ATRA challenge (Fig. 1b) revealed a significant increase in [Ca^2+^]_i_ concurring with toxicity. We thus assessed whether the rise in [Ca^2+^]_i_ levels is causal to toxicity and restrained Ca^2+^ influx from the extracellular environment through silencing STIM1 expression, an activator of the Orai channels facilitating influx of extracellular Ca^2+^ into the cytosol. Decreasing STIM1 expression in these cells led to a robust reduction of ATRA induced toxicity (Fig. 1c). In addition, isradipine, a voltage-gated Ca^2+^ channel (VGCC) inhibitor with neuroprotective potential [29], also rescued cell death in the model (Additional file 3: Figure S2a). Thus, ATRA induced toxicity is reduced by lowering [Ca^2+^]_i_ in this model.

Deregulated kinase activity has been associated with neuronal dysfunction in AD. Thus, [Ca^2+^]_i_ which is a known regulator of mitogen-activated protein kinase (MAPK) signalling [30], may thus mechanistically may underlie the elevated extracellular signal-regulated kinase (ERK1/2) activity observed in preclinical AD models as well as patients [31, 32]. We therefore quantified ERK1/2 activity throughout the ATRA incubation period. The first four days ERK1/2 activity remained relatively low (data not shown), however, the following days, when toxicity started to rise, its activation was found to be significantly increased compared to control conditions (Fig. 1d). To further validate that MAPK ERK deregulation contributes ATRA induced cell death, we applied the MEK inhibitor U0126. Indeed, addition of U0126 counteracted ATRA induced toxicity in a concentration dependent manner (Additional file 3: Figure S2b). This indicates that ERK1/2 signalling, likely as a result of deregulated [Ca^2+^]_i_ observed in the model, plays an important role in mediating cell death as a consequence of ATRA exposure.

Taken together, the model features a rise in [Ca^2+^]_i_ and aberrant downstream signalling (at least ERK1/2) leading to cell death. The assay therefore recapitulates some key characteristics of AD-like neurotoxicity and represents an attractive in-vitro system for identifying mediators and modulators of neurodegeneration.

Subsequently, the model was used to screen library (compiled and provided by reMYND NV) of small molecules for their potential to inhibit toxicity. This led to the identification of structurally highly similar tryptamine-derivatives REM0042826 and REM0043039 (collectively referred to as REM) (Fig. 1e), which potently and concentration dependently lowered cell loss (Fig. 1f and Additional file 1: Figure S1) as well as reducing elevated levels of [Ca^2+^]_i_ (Fig. 1g). In order to emphasise the relevance of the phenotypic screening system described above for identifying novel neuroprotective compounds, we also tested REM in a more conventional AD model involving rat hippocampal neurons challenged with Aβo’s. Incubating the neurons with Aβo’s resulted in pronounced cell death. However, in the presence of REM, the soluble Aβo-elicited cytotoxicity was countered, independently illustrating the compound’s neuroprotective activity (Additional file 4: Figure S3). Altogether, we identified novel compounds that restore Ca^2+^ homeostasis through which they appear to convey their protective effects in cellular models of AD.

### Binding of REM in the prenyl-binding pocket of Pde6δ is required for mitigating toxicity

**Fig. 2:**
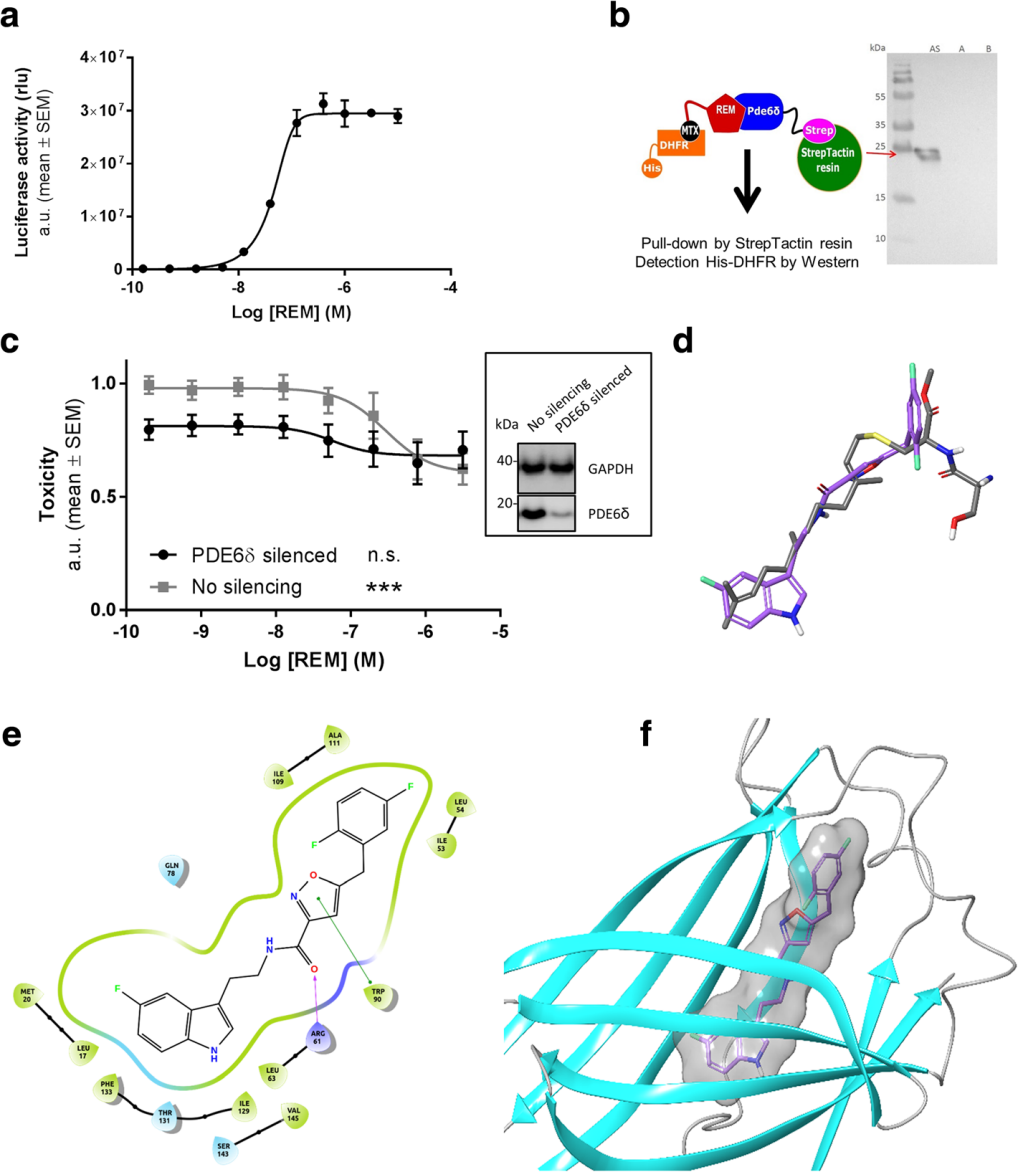
REM bind to the prenyl-binding pocket of Pde6δ. **a** Concentration-dependent interaction of methotrexate fused REM compound (REM0044931) to full-length Pde6δ in the mammalian 3-hybrid assay MASPIT (EC_50_ = 45 nM; *n* = 3). **b** Pde6δ-Strep and REM compound interaction assay. *Left*, schematic representation: *E. coli* lysates producing Pde6δ-Strep, *E. coli* lysates with His-tagged DHFR and 10 μM REM were mixed and subjected to a Strep-Tactin pull-down. *Right,* co-precipitation of DHFR-His was assessed by Western analysis. Pull-down with all compounds as described above (lane AS), without REM-MTX (lane A) or without Pde6δ-Strep (lane B). **c** *Left*, genetic silencing of PDE6δ in the toxicity assay in function of increasing concentrations REM (*n* = 6). Box on the *right*, efficiency of silencing validated by Western blot ((representative immunoblots; immunostaining of GAPDH was determined as a loading control; Pde6δ expression was reduced on average by 88% ± 3%; Average ± SEM) (One-way ANOVA (REM treatment); siNC: *P* = 0.0002; F = 5.29; DF = 7; siPDE6δ: n.s.)). **d** Superimposition of REM compound (purple) and farnesyl (grey). **e** REM docking into the Pde6δ prenyl-binding pocket showing all interacting amino acids. In green indicates hydrophobic amino acids, in dark blue positively charged amino acids and in cyan neutral polar amino acids. **f** REM docked in Pde6δ’s prenyl-binding pocket (ribbon)

Following the identification of the REM, we set out to identify the corresponding target mediating their neuroprotective effects. To this end we coupled REM to methotrexate (resulting in REM0044931, see Methods) and used it as bait in a yeast-based 3-hybrid system [27] to determine interacting proteins using a human cDNA expression library (constructed by Dualsystems Biotech). This led to the identification of several cDNA fragments encoding (parts of) Pde6δ. We independently confirmed the interaction of the compound with full length human Pde6δ in MASPIT [26], a mammalian cell based 3-hybrid assay, and revealed a concentration-dependent interaction with a half-maximal concentration for binding of 45 nM (Fig. 2a). A direct interaction between REM and Pde6δ was validated further in a cell free context by co-immunoprecipitation (Fig. 2b).

In order to confirm that Pde6δ mediates the neuroprotective properties of REM, we silenced PDE6δ in the toxicity cell model (Fig. 2c). Silencing reduced cell death, indicating that Pde6δ mediates (at least in part) toxicity in the model. Although the remaining toxicity in silenced cells was slightly reduced by the compound, an effect which is presumably due to a residual Pde6δ presence, it was not decreased below the level obtained with REM in non-silenced control cells. Thus, the toxicity-mitigating effect of REM requires the presence of Pde6δ.

To better understand the interaction between REM and its target we aligned the cDNA’s identified in the three-hybrid screens. This revealed that the minimal region of Pde6δ required for interaction with REM corresponds to a part of its prenyl-binding pocket (data not shown). The latter hydrophobic cavity accommodates fatty acid chains of small GTPases and is necessary to extract and transport these between cellular membranes [33–35]. In-silico modelling then further elucidated that the identified compounds align closely with prenyl-chains (Fig. 2d). This common topology raised the possibility that it was possible for REM to fit inside Pde6δ’s hydrophobic pocket. Using the cavity’s position to define the docking search space, we assessed whether REM interacts with the pocket. Validation of the docking procedure came from re-docking the farnesyl moiety into the Pde6δ structure. The best docked pose showed a root-mean-square deviation of less than 0.5 Å (all heavy atoms) compared to the published X-ray farnesyl position (Additional file 5: Figure S4a). Taken together, docking of REM revealed that the compound fits well and with high affinity in this cavity (Fig. 2ef and f).

Closer analysis found that the indole group extends deep into the pocket overlapping with the dimethylallyl end of farnesyl and that the indole nitrogen atom is oriented toward Ile129 and Thr131, creating weak interactions, while the amide oxygen atom is engaged in a hydrogen bond with the Arg61 side chain. Furthermore, REM’s oxazole ring forms a pi-pi stacking with Trp90 and the di-fluoro phenyl group occupies the highly hydrophobic entrance of the pocket. The modelling data thus indicate that REM can bind into the prenyl-binding cavity and as a result may prevent Pde6δ’s prenylated substrates from interacting with it.

### REM binding to Pde6δ disrupts the interaction with Rap1 GTPase required for lowering [Ca^2+^]_i_ and toxicity

**Fig. 3:**
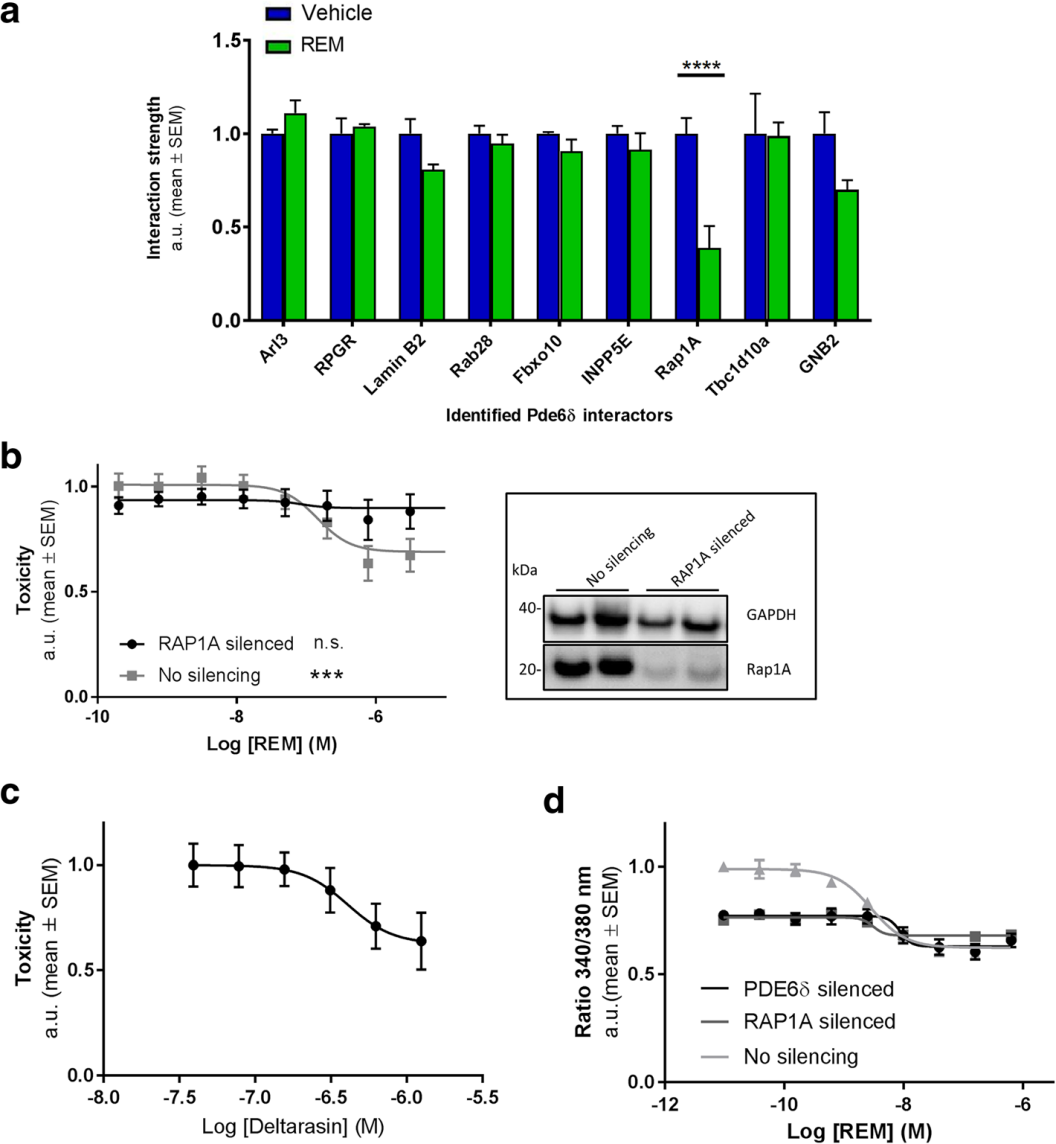
REM reduces toxicity and [Ca^2+^]_i_ by selectively abrogating the interaction of Rap1 with Pde6δ. **a** Affinity purification coupled with mass spectrometry assay to identify interactors associating with Pde6δ in a REM dependent fashion. Signals for each interactor were normalised to the vehicle condition (n = 3; Treatment: *P* = 0.01; DF = 1; F (1, 32)=7.508; Rap1A: *P* < 0.0001; *t* = 5.284; DF = 32). **b** *Left,* the impact of genetic silencing of RAP1A in the toxicity assay in function of increasing concentrations REM (*n* = 6). Box at the *right,* efficiency of silencing validated by Western analysis (representative immunoblots; immunostaining of GAPDH was determined as a loading control; Rap1A expression was reduced on average by 66% ± 8%; Average ± SEM). (One-way ANOVA (REM treatment); siNC: *P* = 0.0004; F = 4.615; DF = 8; siRAP1A: n.s.). **c** Concentration-response curve of deltarasin in the toxicity assay (EC_50_ = 480 nM; *n* = 3). **d** The impact of genetic silencing of PDE6δ or RAP1A in the neuroblastoma cells on cytosolic Ca^2+^ levels in the toxicity assay in function of increasing REM concentrations (*n* = 2 per concentration; one representative experiment of three is shown)

Since the modelling data indicated that REM might compete for Pde6δ substrate binding, we set out to identify such anticipated interactor(s) in an affinity purification coupled with mass spectrometry assay [36] (Fig. 3a) and assessed whether these interactions are impacted by REM. In absence of the compound, a host of previously described Pde6δ binding partners were identified, including Arl3 and RPGR [33, 37, 38] as well as apparently novel interactors like Lamin B2 and Fbxo10. Also, Rab28 and Rap1A representing prenylated GTPases were found to interact with Pde6δ. Importantly, of all detected interactions, only the Pde6δ-Rap1A interaction was significantly and robustly reduced by REM.

Moreover, disruption of the Pde6δ-Rap1A interaction was found to be specific as the interaction with non-prenylated binding partners of Pde6δ such as Arl3 and RPGR, remained unchanged. In fact, there appears to be further specificity regarding the nature of the poly-isoprene lipid group, illustrated by the distinct effects on Pde6δ substrates Rap1A and Rab28. In the presence of REM, Pde6δ’s interaction with geranylgeranylated (C20) Rap1A is inhibited, contrary to that with farnesylated (C15) Rab28. This selectivity of the compound may be a reflection of the intrinsically higher affinity of Pde6δ towards farnesyl over geranylgeranyl [39]. Thus, the experimental data confirmed the *in-silico* docking results and revealed that REM binds to and occludes Pde6δ’s hydrophobic pocket, preventing the interaction specifically with a geranylgeranylated substrate which we identified as Rap1A.

From these data we envisaged that the toxicity-lowering effects of REM are brought about by abrogating the interaction of Rap1A with Pde6δ. To test this hypothesis, we assessed whether toxicity in our model required Rap1A. Silencing RAP1A completely prevented any REM compound induced decrease in toxicity (Fig. 3b), indicating that REM requires Rap1A for its effect. We independently confirmed this result by using deltarasin, a compound structurally unrelated to REM, which also binds in Pde6δ’s prenyl-binding pocket [40] and, similarly to REM, showed a trend towards counteracting cell-death in our model of neurotoxicity (Fig. 3c).

Having established that REM targets the Rap1-Pde6δ interaction for lowering neuronal toxicity in our model, we tested whether decreasing elevated [Ca^2+^]_i_ requires both proteins. To this end, we measured [Ca^2+^]_i_ levels in Pde6δ and Rap1A silenced cells in function of REM treatment in the neurotoxicity model. This revealed that silencing of both PDE6δ and RAP1A reduced [Ca^2+^]_i_ levels to a similar extent (Fig. 3d), indicating that both Pde6δ and Rap1A are required for elevated [Ca^2+^]_i_ in the model. Furthermore, in both Pde6δ and Rap1A silenced cells, the already low levels of [Ca^2+^]_i_ were not reduced below the levels recorded in non-silenced cells by REM. Thus, the REM mediated decrease of [Ca^2+^]_i_ in the model is, like toxicity, dependent on Pde6δ and Rap1A.

Taken together, we revealed geranylgeranylated Rap1A as a novel substrate of Pde6δ’s prenyl-binding pocket and that this interaction is involved in Ca^2+^ dyshomeostasis and associated cytotoxicity.

### Disruption of the Pde6δ-Rap1 interaction has distinct effects on spatially discrete Rap1-functions

Pde6δ is known to act as a cytosolic chaperone controlling the subcellular membrane localisation (and thereby their function) of prenylated substrates [25, 34, 37, 41], such as Rap1. In particular, Rap1 was shown to regulate signalling of MAPK-ERK1/2 at spatially discrete subcellular locations [42–46]. In resting neurons for instance, basal levels of Ca^2+^ and cAMP [47] control a membrane pool of Rap1-ERK1/2, which phosphorylates (inactivates) protein Kv4.2, the main A-type K^+^ channel driving the fast transient outward K^+^ current (I_A_) involved in action potential AHP and repolarisation in pyramidal neurons [48]. On the other hand, upon depolarisation a different subcellular pool of Rap1-ERK1/2 has been implicated in signalling towards nuclear targets mediating gene transcription [49], whereas an exclusively intracellular pool of mGluR-Rap1-ERK1/2 mediates the internalisation of surface AMPA receptors during LTD [50–52].

Given that Pde6δ regulates the subcellular membrane localisation of prenylated substrates, we hypothesised that Pde6δ mediated control over Rap1 functions at spatially discrete pools specifies its signalling outcome. Accordingly, disruption of the Rap1-Pde6δ interaction by REM should alter activity of distinct ERK1/2 signalling pathways. To test this hypothesis, we assessed basal and mGluR driven Rap1-ERK1/2 activity.

**Fig. 4:**
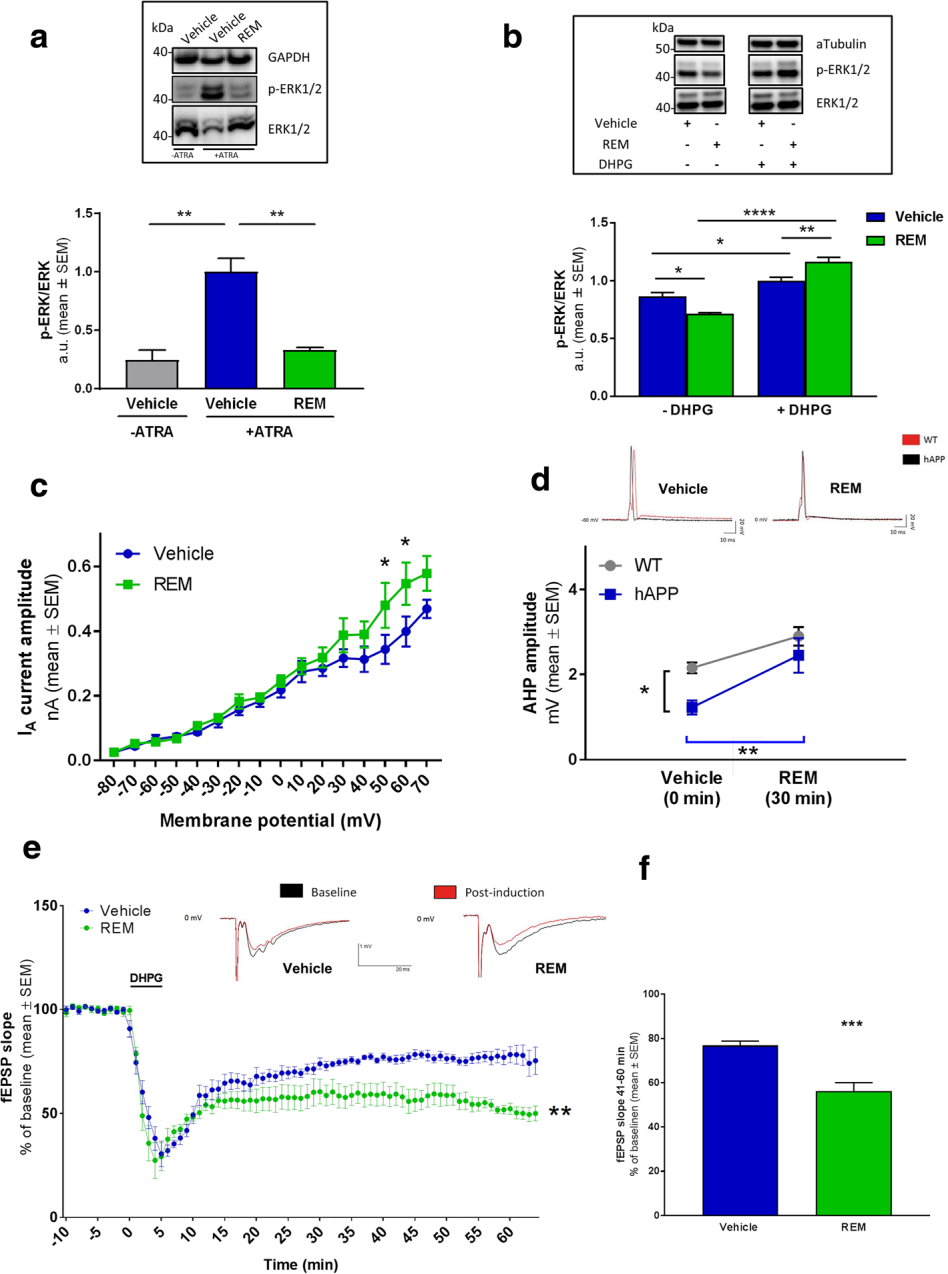
REM modulates spatially discrete Rap1-ERK1/2 signalling processes. **a** Western blot analysis of ERK1/2 phosphorylation in neuroblastoma cells after 7 days with or without ATRA toxicity challenge and with or without (0.25 μM) REM (*n* = 3; Vehicle +/− ATRA: *P* = 0.0066; *t* = 5.191; DF = 4; Vehicle/REM + ATRA: *P* = 0.0052; *t* = 5.55; DF = 4). Representative Western blots are shown in inset above the graph. **b** Western blot analysis of ERK1/2 phosphorylation ratio to total ERK1/2 in DIV ≥19 mouse primary hippocampal neurons treated with or without 50 μM DHPG and REM/Vehicle as indicated (*n* = 6; ordinary two-way ANOVA followed by Tukey’s multiple comparison test; *P* < 0,0001; DF = 1; F (1, 20) = 27,41; vehicle +/− DHPG: *P* = 0,0248; q = 4,431; DF = 20; REM +/− DHPG: *P* < 0.0001; q = 14,9; DF = 20; vehicle/REM - DHPG: *P* = 0,0107; q = 4,975; DF = 20; vehicle/REM + DHPG: *p* = 0,0047; q = 5,497; *DF* = 20). Representative Western blot images are shown in insets above graph. **c** K^+^ current analysis in hAPP mice treated with a single dose of REM or vehicle show that application of REM increases the I_A_ contribution at higher membrane potentials (50–60 mV) (n = 7 per condition; RM-two way ANOVA Interaction: *P* = 0.056; DF = 15; F(15,180) = 1.691; Sidak’s multiple comparison: membrane potential 50 mV Vehicle/REM: *P* = 0.0326; *t* = 3.122; DF = 192; membrane potential 60 mV Vehicle/REM: *P* = 0.0130; *t* = 3.401; DF = 192). **d** Analysis of AHP amplitudes in function of REM treatment obtained by longitudinal somatic recordings of single APs in WT and hAPP slices that were incubated with vehicle or 2 μM REM. (Two-way ANOVA repeated measures, followed by Sidak’s multiple comparison test; n = 7 WT mice or *n* = 8 hAPP mice; Treatment: *P* = 0.0015; DF = 1; F (1, 13)=15.91; Genotype: *P* = 0.0279; DF = 1; F (1, 13)=6.127; WT/APP + Vehicle: *P* = 0.0376; *t* = 2.502; DF = 26; hAPP + Vehicle/REM: *P* = 0.006; *t* = 3.635; *DF* = 13.) Examples of recorded traces are shown above the graph. **e** CA3-CA1 LTD: quantification of fEPSP slopes from hippocampal slices. Slices were continuously perfused with artificial CSF containing vehicle or REM for (at least) one hour prior to (pre-incubation), as well as during and after the addition of DHPG (two-way ANOVA repeated measures; *n* = 6 mice per condition; Treatment: *P* = 0.0032; DF = 1; F (1, 10)=14.91). Example traces are shown above the graph. **f** Graph shows the summary of mean fEPSP slopes during the 41–60 min interval from **d** (*n* = 6 mice per condition; *P* = 0.0008; *t* = 4.725; DF = 10)

As shown previously in the toxicity model, basal ERK1/2 activity was found to be strongly increased under conditions of cell death (Fig. 4a). However, in the presence of REM, this ERK1/2 over-activation was strongly reduced (Fig. 4a). Also, in hippocampal neurons, where synaptic activity was eliminated using the Na^+^ channel blocker TTX, REM treatment led to a reduction in basal ERK1/2 activity (Fig. 4b). Conversely, upon stimulation with the selective group 1 mGluR agonist DHPG the fold Rap1-ERK1/2 activation in cells incubated with REM was increased compared to vehicle treated cells (Fig. 4b). Collectively, these results indicate that the basal activity of Rap1-ERK1/2 signalling in resting cells, which is considered to represent the membrane-associated pool [49], is decreased by REM, while activation of an intracellular DHPG responsive pool [53] of Rap1-ERK1/2 signalling is increased.

We then tested whether such REM-induced changes on different pools of Rap1-ERK1/2 resulted in functional changes in an AD model. Previous studies have shown that lowering basal Rap1-ERK1/2 signalling reduces the phosphorylation of plasma membrane Kv4.2 channels and thereby increases neuronal repolarisation [49, 54]. This regulation appears highly relevant for AD because in Aβ_1–42_ exposed hippocampal primary neurons and in numerous human transgenic APP mouse models the activity of Kv4.2 is reduced [10, 55]. As a result of this, transgenic APP models were shown to feature an A-type K^+^ channel-deficiency driving neuronal hyperactivity [10, 56] and therefore we anticipated REM to mitigate this phenotype. To test this, we quantified the I_A_ in function of REM treatment. Hippocampal CA1 somatic voltage-clamp recordings were performed in acute slices excised from human transgenic APP[V717I] (hAPP) [21] mice orally administered with vehicle or a single dose of REM at 30 mg/kg. The recorded I_A_ current (as isolated by subtraction of the 4-AP insensitive from the total K+ current (Additional file 6: Figure S5a)) was significantly increased at higher depolarising pulses by REM compared to the vehicle group (Fig. 4c).

Furthermore, to test the impact of REM on neuronal repolarisation, single action potentials (AP) were assessed by somatic patch-clamp of hippocampal CA1 pyramidal neurons from acute brain slices of hAPP and WT mice. These recordings revealed that the AHP amplitude was significantly decreased in hAPP mice compared to WT mice, (Fig. 4d). After these baseline measurements, treatment of the same hAPP slices with REM significantly increased (normalised) the AHP amplitude (Fig. 4d). No significant impact of REM was observed on other AP parameters in hAPP slices (data not shown). Since Kv4.2 plays a key role in regulating dendritic AP shape and propagation, we also assessed CA1 dendritic single AP’s (recorded > 200 μm from soma) in hAPP acute brain slices. These recordings likewise revealed that REM robustly increased the AHP amplitude (Additional file 6: Figure S5b).

Collectively, these data indicate that REM predominantly facilitates the K^+^ channel driven component of AP’s. This is consistent with the hypothesis that lowering basal Rap1-ERK1/2 signalling by REM treatment increases K^+^ channel activity and neuronal repolarisation.

In addition, we assessed the functional impact of a REM-driven increase in intracellular mGluR5-Rap1-ERK1/2 signalling (Fig. 4b) and ensuing LTD. As expected, DHPG induced a significant and robust decrease in fEPSP slopes compared to baseline, indicating a depression of synaptic efficacy through AMPA receptor removal (Fig. 4d) [57]. Pre-incubation with REM led to a significantly greater reduction in fEPSP slopes 40 min after the induction compared to vehicle (Fig. 4d and e), revealing that mGluR driven LTD is enhanced by the compound. Thus, these data indicate that Pde6δ controls intracellular Rap1-ERK1/2 signalling regulating synaptic plasticity.

Altogether, we demonstrated that Pde6δ modifies the outcome of different subcellular Rap1-ERK1/2 signalling pathways. Accordingly, REM, by neutralising Pde6δ’s control over Rap1, reduces basal Rap1-ERK1/2 activity in resting neurons, which enhances the K^+^ channel driven component of AP’s; while increasing mGluR-Rap1-ERK1/2 signalling, leading to greater synaptic depression.

### Abrogation of the Rap1-Pde6δ interaction by REM reduces VGCC activity

**Fig. 5:**
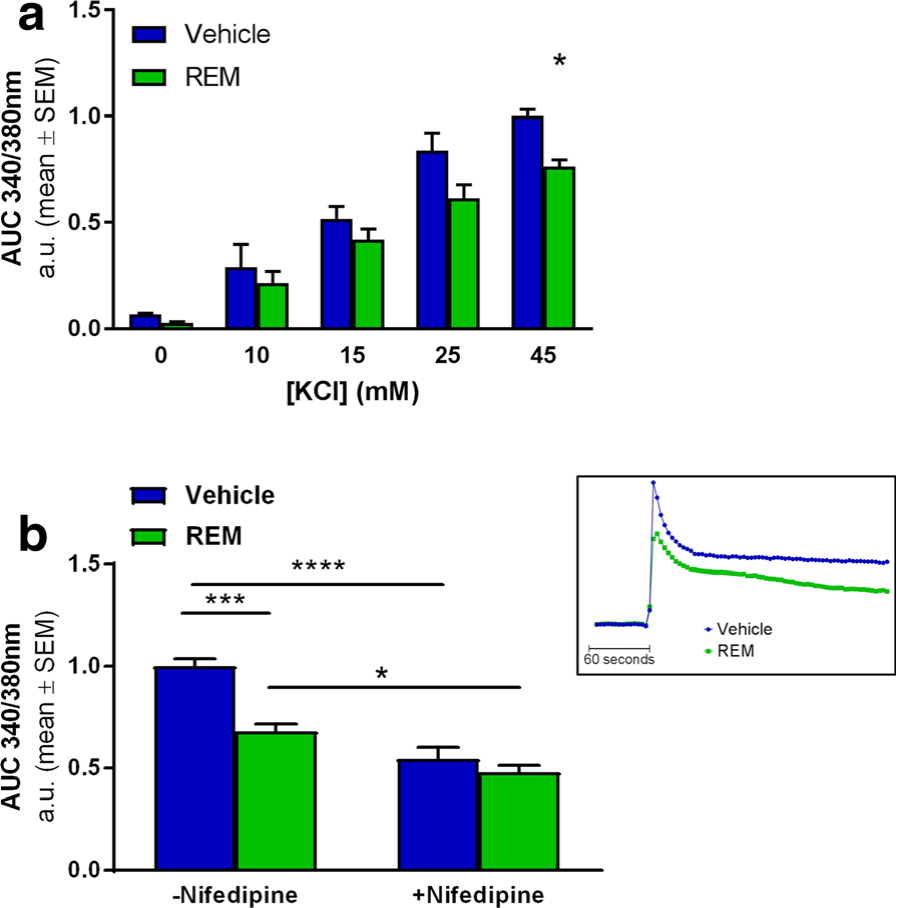
REM represses VGCC activity in primary hippocampal neuron cultures. **a** Quantification of [Ca^2+^]_i_ influx using Ca^2+^ indicator Fura-2 after KCl instigated depolarisation at increasing concentrations in function of 1.5 μM REM treatment. Graphs represent the normalised mean Area’s Under the Curve’s (AUC) over a 4 min period after the addition of KCl (*n* = 3; Treatment: *P* = 0.0015; DF = 1; F (1, 20)=13.47; KCl concentration: *P* < 0.0001; DF = 4; F (4, 20)= 68.45; Multiple comparisons test: 45 mM KCl Vehicle/REM: *P* = 0.0454; *t* = 2.881; DF = 20). **b** AUC’s are determined as in (a) after exposure to 45 mM KCl in presence or absence of 12.5 μM nifedipine as indicated. A representative Ca^2+^ trace (+/−REM) is depicted in the small inset next to the graph. (*n* = 5; REM Treatment: *P* = 0.0003; DF = 1; F (1, 16)=20.87; +/− Nifedipine: *P* < 0.0001; DF = 1; F (1, 16)=60.32; Interaction: *P* = 0.008; DF = 1; F(1.16) = 9.156; Multiple comparisons test: Vehicle/REM – nifedipine: P = 0.0004; *t* = 5.37; DF = 16; Vehicle +/− nifedipine: *P* < 0.0001; *t* = 7.631; DF = 16; REM +/− nifedipine: *P* = 0.0241; *t* = 3.352; DF = 16)

Previous studies demonstrated that Rap1-ERK1/2 signalling regulates the surface expression and consequently the activity of L-type VGCC [58]. We therefore tested whether inhibiting Pde6δ’s control over Rap1 alters the activity of VGCC’s, measuring Ca^2+^ influx after depolarisation induced by high extracellular K^+^ in function of REM treatment. Here, the addition of potassium chloride (KCl) to the extracellular medium produced a concentration-dependent increase in [Ca^2+^]_i_ (Fig. 5a). This Ca^2+^ influx was shown to be in part mediated by L-type VGCC’s, since the influx was reduced by approximately 50% in the presence of L-type channel specific inhibitor nifedipine (Fig. 5b). Incubation with REM significantly lowered Ca^2+^ influx only at KCl concentrations above 25 mM (Fig. 5a), indicating that REM specifically inhibits VGCC’s that are active during relatively strong depolarisations such as L-type channels [59].

Co-application of nifedipine together with REM produced no additional inhibition compared to nifedipine alone, indicating that the compound acts on nifedipine sensitive channels (Fig. 5b and Additional file 7: Figure S6). Thus these data show that Rap1-ERK1/2 signalling controls VGCC surface activity and consequently neurotransmitter release upon depolarisation [58]. Further we show that Pde6δ modifies Rap1 mediated VGCC activity.

### Inhibition of the Pde6δ-Rap1 interaction with REM restores spike frequency adaptation in hAPP mice

**Fig. 6:**
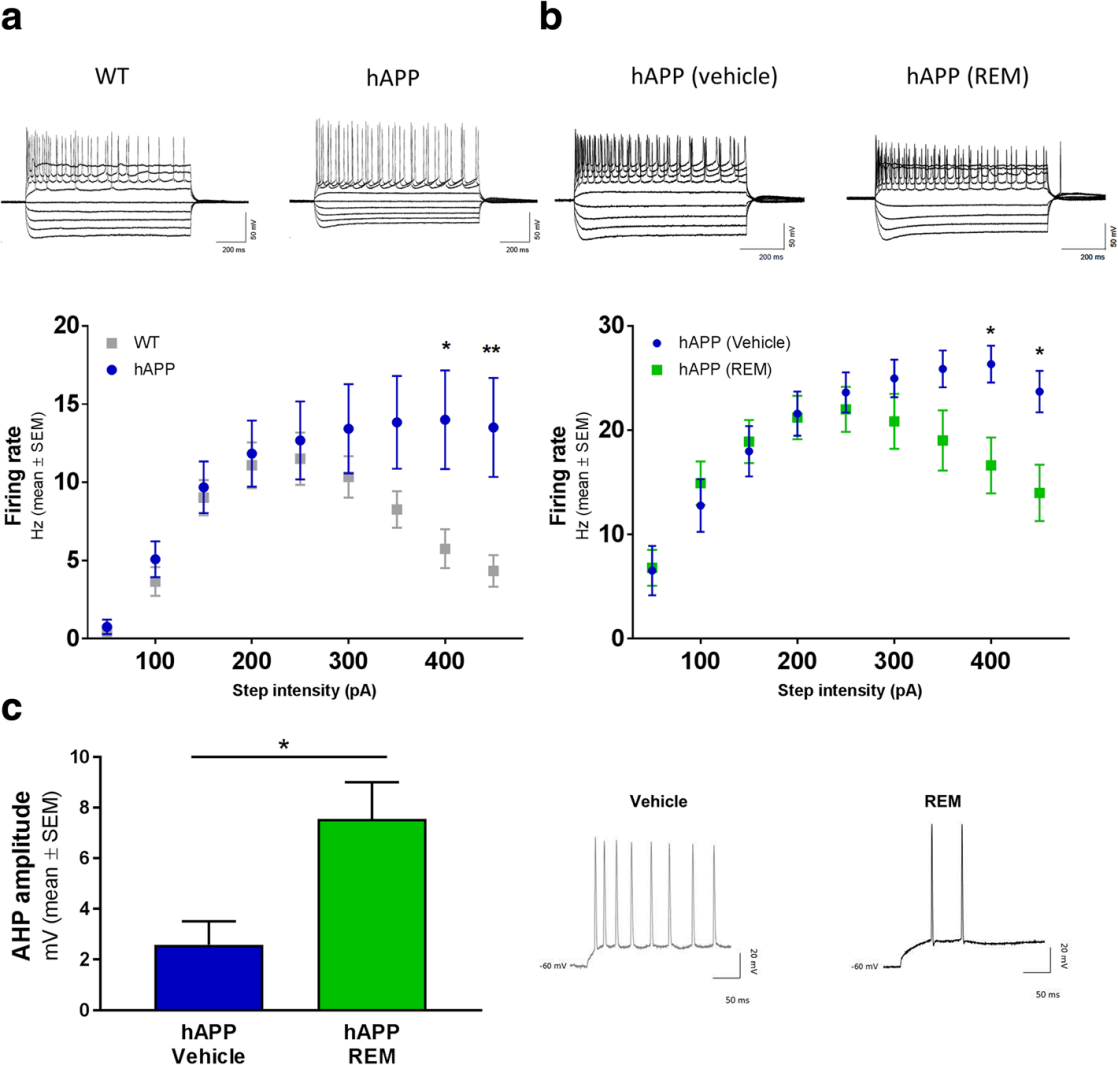
hAPP deficit in spike frequency adaptation is rescued by REM. **a** Dendritic firing rate of hippocampal CA1 pyramidal neurons in WT and hAPP slices in response to increasing depolarising step current injections (50–450 pA; *n* = 12 mice per genotype; repeated measures two-way ANOVA: Interaction: *P* = 0.0006; DF = 8; F(8,176) = 3.649; Sidak’s multiple comparison: 400 pA: *P* = 0.0218; *t* = 3.069; DF = 198; 450 pA: *P* = 0.0071; *t* = 3.41; DF = 189). **b** Dendritic firing rate of hippocampal CA1 pyramidal neurons in WT and hAPP slices perfused with either vehicle or 2 μM REM in response to depolarising step current step injections (50–450 pA). (Vehicle: *n* = 12 mice; REM: *n* = 15 mice; repeated measures two-way ANOVA: Interaction: *P* < 0.0001; DF = 8; F(8,200) = 5.082; Sidak’s multiple comparison: 400 pA: *P* = 0.0168; *t* = 3.146; DF = 225; 450 pA: *P* = 0.0079; *t* = 3.372; DF = 225). Note that the overall firing rates were increased in the second experiment (b) compared to the first one (a). This is likely due to the addition of the vehicle DMSO which was shown previously to enhance neuronal activity [60, 61]. **c** Dendritic AHP amplitude in CA1 neurons in response to the first depolarising current that induced an AP in hAPP slices with or without REM (Vehicle: n = 12 mice; REM: *n* = 16 mice; *P* = 0.0128; *t* = 2.673; DF = 26)

Our data thus far demonstrated that REM facilitates neuronal repolarisation, decreases synaptic efficacy and lowers VGCC activity, each process on its own geared towards restraining neuronal excitability under conditions of strong stimulation. We therefore assessed the neuronal firing rate in mouse hippocampal CA1pyramidal cells by apical dendritic current-clamp. Thus, in an initial experiment we set out to compare WT mice with their hAPP transgenic counterparts. The results show that in WT mice, the firing rate increased with each current step intensity until 250 pA, after which the firing frequency started to decline. This decline at high depolarising currents indicates a functional spike frequency adaptation process, a natural safety mechanism protecting neurons from overstimulation. Contrary to what was observed in WT cells, neurons from hAPP mice did not demonstrate a decrease in firing frequency at depolarising currents above 250 pA, meaning that mechanisms underlying spike frequency adaptation in these neurons are dysfunctional (Fig. 6a).

Having shown that overexpression of mutant hAPP led to enhanced neuronal activity, we conducted a second experiment to assess the impact of REM on the neuronal firing rates in these mice. Application of REM restored spike frequency adaptation in hAPP mice as opposed to vehicle treatment (Fig. 6b).It has to be noted however that the addition of the vehicle DMSO led to an overall increase in firing rate, an effect that has been previously documented [60, 61]. This action of DMSO is further supported by the lack of action potentials generated at the lowest injected current (50 pA) in absence of vehicle (Fig. 6a) contrary to their presence following DMSO treatment (Fig. 6b). Nevertheless, regardless of this DMSO effect, the spike frequency adaptation process in hAPP mice was restored by REM treatment.

In-depth analysis of action potentials evoked at the first depolarisation current (50 pA) revealed a significant increase in the AHP amplitude in hAPP neurons treated with REM (Fig. 6c) confirming the single AP findings. Altogether, our data demonstrate that REM normalises spike frequency adaptation in hAPP CA1 neurons and increases their repolarisation as evidenced by enhanced AHP.

### Modulation of the Pde6δ-Rap1 interaction and ERK1/2 signalling by REM decreases phosphorylation of tau and rescues behavioural deficits in different mouse models for AD

**Fig. 7:**
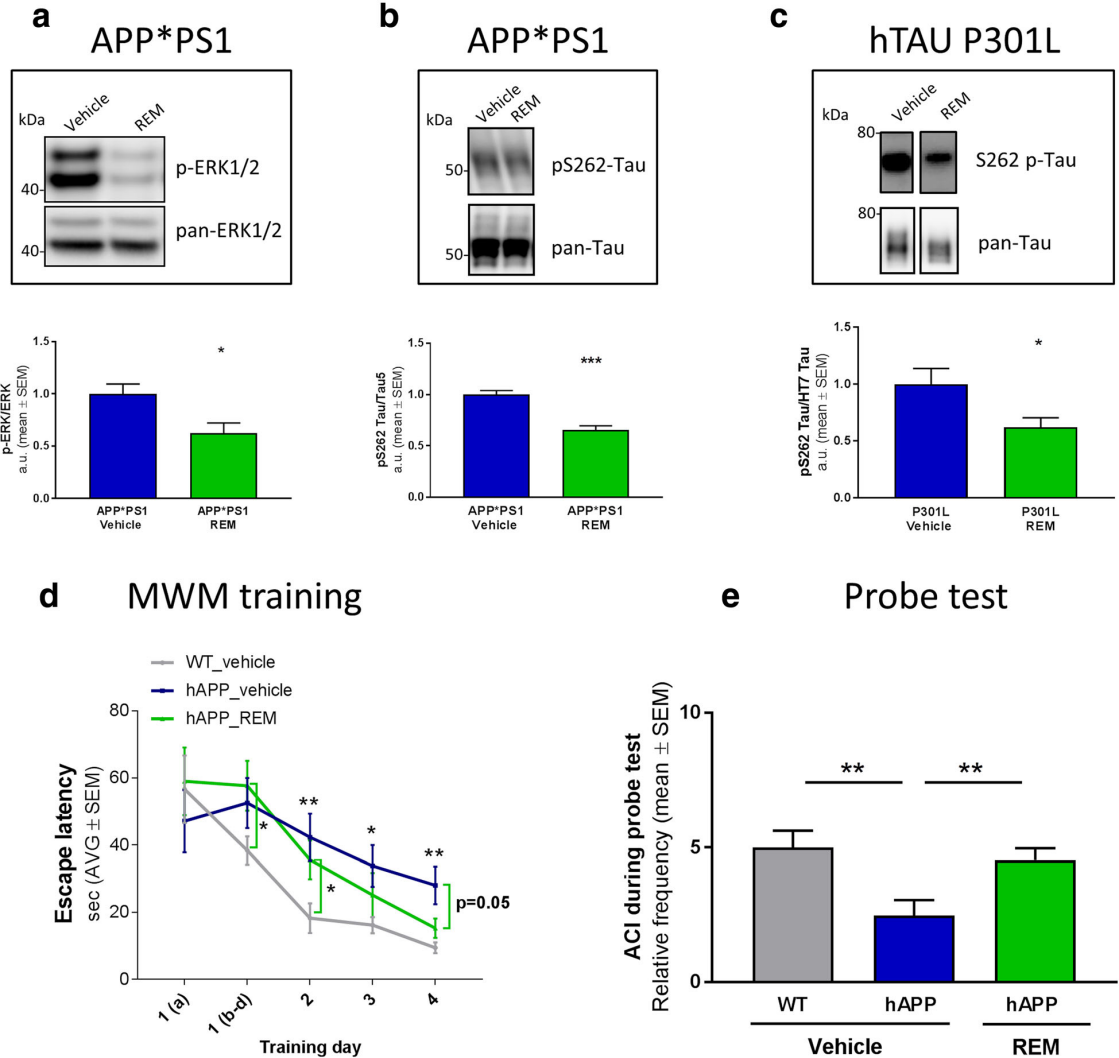
REM treatment reduces ERK1/2 signalling, Tau phosphorylation and cognitive impairments in mouse models of AD. **a** Western blot analysis quantifying p-ERK1/2 ratio to total ERK1/2 (*n* = 6 vehicle or 5 REM treated mice; *P* = 0.0220; *t* = 2.763; DF = 9) or **b** phosphorylated Tau at epitope: pS262 ratio to total tau (Vehicle: *n* = 4 mice; REM: n = 5 mice; *P* = 0.0007; *t* = 5.735; DF = 7) in cortex of transgenic hAPP*PS1 mice treated for 2 weeks with vehicle or REM0043039. Representative immunoblots are shown in inset above the graph. **c** REM significantly decreases pathological tau phosphorylation on epitope S262 compared to total tau also in the brainstem of tau P301L transgenic mice (*n* = 9 vehicle and 13 REM treated mice; *P* = 0.0217; *t* = 2.491; DF = 20). **d** Escape latency during MWM training comparing WT to hAPP mice with or without REM (*n* = 12 mice per condition; t-test: WT Vehicle/hAPP Vehicle: day 2 *P* = 0.0083; *t* = 2.901; DF = 22; day 3 *P* = 0.0153; *t* = 2.629; DF = 22; day 4 *P* = 0.00431; *t* = 3.183; DF = 22; hAPP Vehicle/REM: day 4 P = 0.056; *t* = 2.015; DF = 22; WT Vehicle/hAPP REM: day 1(b-d) *P* = 0.034; *t* = 2.256; DF = 22; day 2 *P* = 0.0264; *t* = 2.381; DF = 22 **e** Graphs showing the annulus crossing index (ACI) representing the number of crossings over the platform site in the target area adjusted for crossings over corresponding areas in other quadrants during the probe test (*n* = 12 mice per condition; ACI: Vehicle WT/ Vehicle hAPP: *P* = 0.0069; *t* = 2.98; DF = 22; hAPP Vehicle/REM: *P* = 0.0098; *t* = 2.829; DF = 22)). (One data-point from hAPP vehicle group is below 0 and is included in the analysis but not shown in the graph)

We showed earlier that abrogation of the Pde6δ-Rap1 interaction by REM reduced [Ca^2+^]_i_ and ERK1/2 signalling in resting neurons. Since MAPK are important Tau kinases involved in hyperphosphorylation of the protein in an AD context, REM treatment is expected to lower pathological Tau phosphorylation. Hence, in-vivo efficacy of REM was assessed in hAPP*PS1 transgenic mice [22] and two different hTAU transgenic mouse strains [62, 63]. Following two weeks sc administration of REM to hAPP*PS1 mice, Western blot analysis of brain extracts revealed a significant decrease in ERK1/2 activity (Fig. 7a), similar to the *in-cellulo* observations presented earlier (Fig. 4a). Moreover, pathological phosphorylation of endogenous Tau on different epitopes was reduced (pS262 epitope; Fig. 7b; AT8, pT231 and AD2 data not shown). Similarly, REM treatment of hTAU-P301L or hTAU-R406W transgenic mice resulted in a significant decrease in the phosphorylation of different epitopes of Tau (pS626-Tau, hTau-P301L: Fig. 7c; pS202 and pT205, hTau-R406W: Additional file 8: Figure S7a). No impact was observed on total levels of Tau protein. As such, it is reasonable to assume that, since MAPK’s are known to be involved in Tau phosphorylation [31], the reduction of the latter is a downstream effect of REM’s action on ERK. Nevertheless, we cannot exclude the contribution of other Ca^2+^-dependent Tau kinases at this point.

Finally, we set-out to assess cognitive deficits in AD mice in function of REM treatment. To this end, hAPP and WT mice were tested in a MWM paradigm to assess (hippocampal based) spatial learning and memory after chronic REM or vehicle treatment. Following four days of training, in which the REM treated hAPP mice showed a reduced escape latency (Fig. 7d) and search path (Additional file 9: Figure S8a). During the probe task on day five, REM treated hAPP mice demonstrated a fully normalised cognitive performance, similar to that of WT mice (Fig. 7e), as opposed to the vehicle treated ones. After completion of the probe test, pharmacologically relevant exposure of REM in the brain of treated animals was confirmed by liquid chromatography-tandem mass spectrometry (Additional file 9: Figure S8b). Furthermore, although no MWM-based learning deficit was observed in R406W-Tau mice compared to WT mice (data not shown), the transgenic mice showed a significantly reduced mobility (swim speed), which may represent a motoric deficit observed in most transgenic Tau models as a consequence of Tau pathology in the hind brain. This motoric deficit was fully normalised after a four weeks treatment with REM (Additional file 8: Figure S7b). In addition, neither short-, nor long-term treatment with REM at the predetermined effective dose led to any side effects in the mice.

Altogether, these data demonstrate that, by modulation of the Pde6δ-Rap1 interaction, REM treatment reduces phosphorylation of protein Tau and rescues behavioural deficits in different AD-like mouse models.

## Discussion

We report the development of a cell-based assay of neuronal degeneration featuring elevated levels of [Ca^2+^]_i_, deregulation of intracellular signalling and cytotoxicity. As such the model recapitulates key aspects of neurodegeneration in AD and represents a promising system for assessing underlying disease mechanisms and for identifying new therapies. To this end we have discovered novel compounds (REM), which selectively abrogate the binding of Pde6δ to small GTPase Rap1, thus representing a valuable pharmacological tool to study this interaction in more detail. Analysis of REM activity revealed that its target Pde6δ -by controlling Rap1- impacts Ca^2+^ homeostasis, plasticity and excitability, processes fundamentally underlying neuronal function and survival. Accordingly, normalising these processes by REM treatment mitigates neuronal toxicity, reduces in-vivo Tau phosphorylation and improves cognitive performance in animal models of AD.

**Fig. 8:**
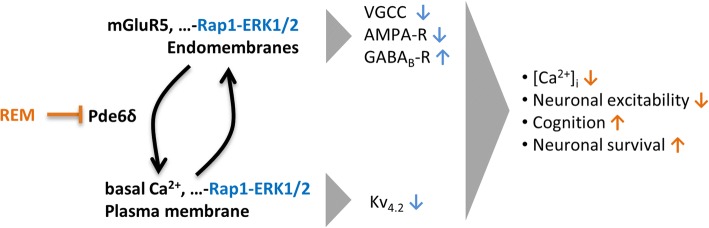
Model illustrating how Pde6δ links different pools of Rap1 to Ca^2+^ homeostasis, neuronal excitability, cognition and neuronal survival. The Rap1-ERK1/2 cascade emanating from endomembranes represses VGCC and AMPA-R but increases GABA_B_ receptor activity, thus favouring a reduction in neuronal activity. On the other hand, a plasma membrane associated pool of basal Rap1-ERK1/2 activity inhibits Kv4.2 channels, which promotes neuronal activity. Assuming that like for Ras [41], removal of Rap1 from the plasma membrane is endocytosis dependent, neuronal activity coinciding with endocytosis will redistribute Rap1 towards endomembranes. This Rap1 redistribution is geared towards lowering neuronal excitability, it could represent a natural feedback control to keep neuronal activity and VGCC mediated Ca^2+^ influx within certain limits and protecting neurons. Rap1 recycling from endomembranes to the plasma membrane is facilitated by Pde6δ. Thus, inhibition of Rap1 plasma membrane recycling by REM augments the small GTPase’s presence and activity at endomembranes, while depleting it from the plasma membrane. As a result, under conditions of persistent excessive neuronal activity (such as in AD and epilepsy), REM rearranges the different pools of Rap1 towards restraining Ca^2+^ influx and neuronal excitability, resulting in neuroprotection. (In blue: Rap1-ERK1/2 actions at different membranes; orange arrows indicate Rap1-ERK1/2 functions altered by REM)

Pde6δ as a GDI-like solubilisation factor facilitates non-vesicular inter-membrane transport of prenylated proteins [64]. As such, Pde6δ ensures the correct spatial organisation of cargo proteins for interaction with their respective effectors and/or modulators and thus determines the functional outcome of their signalling [25, 41]. In contrast to what the somewhat misleading name suggest, Pde6δ has no phosphodiesterase activity but participates in intracellular trafficking of cognate substrates and is more appropriately coined prenyl-binding protein δ (PrBP/δ) [64]. We here provide further evidence that Pde6δ interacts with Rap1 via its prenyl-binding pocket, confirming earlier findings [35]. In case of Ras, Pde6δ was shown to facilitate shuttling between different membrane locations in the cell by shielding the GTPase’s prenyl-chain from the hydrophilic cytosol in its hydrophobic pocket [25, 41]. We therefore propose that, like for Ras, Pde6δ mediates the inter-membrane transport of Rap1 determining its subcellular distribution and consequently its function (Fig. 8).

According to this model, Pde6δ driven transport would enhance Rap1 enrichment at membranes destined for the PM (Fig. 8). The mechanism of Rap1 removal from the PM remains to be determined. Nevertheless, it seems reasonable to assume that it, like for Ras, follows endocytosis [25]. Thus, we envisage that under conditions of elevated neuronal activity and concomitant high rate of endocytosis, transport of Rap1 towards intracellular membranes is enhanced. In other words, in active neurons Rap1 is removed from the PM and concentrated on intracellular membranes. This relocalisation of Rap1 then reconfigures its functions towards more efficient reduction of neuronal excitability. The endomembrane directed transport of Rap1 on the other hand is countered by Pde6δ, which actively enhances the small GTPase’s recycling to the PM. REM antagonises this function of Pde6δ and thereby increases Rap1 on endomembranes at the expense of a PM localised pool, gearing the small GTPase’s functions towards restraining neuronal excitability. Indeed, following high stimulation (electrically or chemically), REM, through blockage of Pde6δ mediated Rap1 recycling, decreases Ca^2+^ influx and excessive neuronal activity (Fig. 8).

Taken together, the subcellular membrane distribution of Rap1 may represent a dynamic auto-regulatory system containing neuronal excitability within physiological limits. During excessive stimulation such control would prevent neuronal hyperactivity and associated excitotoxicity, whereas at the same time keeping neurons sensitive for activation following less strong stimulations. Such a mechanism does not necessarily imply that Pde6δ-Rap1 functionality is altered in or causal to AD. However, under conditions of neuronal hyperexcitability, such as occurring during AD, REM would simply gear Rap1-ERK signalling towards a reduction of excessive activity.

Considering that neuronal activity is closely associated with Aβ production as well as its release and thereby aggregation and spread [65]; we anticipate that REM treatment, by mitigating neuronal hyperactivity, has beneficial effects on the amyloid burden. Although we have not yet addressed this aspect of REM action, it remains an interesting possibility to be explored in future studies.

Moreover, hyperexcitability underlies the susceptibility of hAPP mice to suffer from epileptic seizures [66], a phenomenon which is also observed in AD patients [20]. Here we show that REM by abrogating the Rap1-Pde6δ interaction can normalise neuronal excitability in hAPP mice. This outcome is not unexpected, since many Rap1 functions affected by REM treatment also represent major target classes for known anti-epileptic drugs [67], e.g. voltage-dependent Ca^2+^ and K^+^ channels and ionotropic glutamate AMPA receptor. Moreover, Kv4.2, the main A-type K^+^ channel, appears to be highly involved in the most prevalent form of epilepsy in adults, namely temporal lobe epilepsy [68] and experimental models thereof [69]. We therefore envisage that reducing neuronal hyperexcitability by inhibiting the interaction between Pde6δ and Rap1 has therapeutic potential not only in AD, but also seizure disorders such as epilepsy.

Along the same lines and, an intracellular pool of Rap1 was previously shown to control the surface expression and therefore activity of the inhibitory neurotransmitter receptor gamma-aminobutyric acid receptor B (GABA_B_) [70]. Accordingly, we anticipate that increasing Rap1 on endomembranes by REM promotes GABA_B_ surface expression, providing an additional mechanism of controlling neuronal activity (Fig. 8). As such, it would be interesting to evaluate such a REM effect in follow-up studies.

As discussed above, Rap1-ERK1/2 signalling controls a host of spatially discrete processes underlying neuronal function and survival. Hence under pathological conditions, where cytosolic Ca^2+^ levels are chronically elevated, the ensuing inappropriate activation of basal Rap1-ERK1/2 may lead to a reduction in Kv4.2 activity [49] and thus contribute to the neuronal hyperexcitability observed in the AD models [10, 19]. In agreement with this notion, we showed that REM diminishes basal Ca^2+^ driven Rap1-ERK1/2 signalling originating at the PM and thereby restores AP repolarisation and the AP firing pattern in AD models possibly by increasing the I_A_–type K^+^ current. In addition, REM prevented Ca^2+^ induced chronic ERK1/2 activation, a process which can induce apoptosis [71]. Accordingly, we show that downregulating basal Rap1-ERK1/2 activity, by inhibiting the Pde6δ-Rap1 interaction, is neuroprotective in models of AD. This is in agreement with previous findings where more general ERK1/2 inhibition approaches conferred neuroprotection [72–75].

## Conclusion

The data presented here indicate that Pde6δ is an important regulator of Rap1-ERK1/2 signalling by controlling the latter’s spatial organisation in the cell. Consequently, the newly identified neuroprotective REM compounds, by targeting Pde6δ mediated Rap1 inter-membrane shuttling, restrains critical aspects of neuronal functionality, including Ca^2+^ influx and excitability, which ultimately improve neuronal health and survival under conditions of excessive stimulation. Thus, targeting Pde6δ has promising therapeutic potential for disorders characterised by neuronal hyperactivity, such as AD and epilepsy.

## Additional files

Additional file 1: **Figure S1.** ATRA induced cell death in the model is enhanced by expression of a cDNA encoding mutant human Tau and is prevented by REM. **a** Left: Western blot analysis confirmed successful tau overexpression (approximately 2-fold) in neuroblastoma cells containing the hTAU-P301L plasmid. Right: Toxicity was determined by quantifying LDH release in the medium in BE (2)-M17 neuroblastoma cells with (hTAU-P301L) and without (Control) expression of a cDNA encoding hTAUP301L incubated for 7 days with or without ATRA. The ATRA induced toxicity in hTAU-P301L cells represents the “toxicity assay” used in all following experiments (two-way ANOVA: +/− ATRA: *P* < 0.0001, F(1, 36)=4276, DF = 1; +/− hTAU-P301L: *P* < 0.0001, F (1, 36)=255.3, DF = 1; Sidak’s multiple comparison test: control +/− ATRA: P < 0.0001, *t* = 27.54, DF = 36; hTAU-P301L +/− ATRA: *P* < 0.0001, *t* = 64.93, DF = 36; *n* = 10). # denotes the effect of hTAU-P301L expression silencing compared to control cells, * indicates the effect of ATRA treatment. **b** Representative images of neuroblastoma cells exposed to ATRA for six days treated with either REM or vehicle. **c** Treating hTAU-P301L cells in the toxicity assay with tau aggregation inhibitior methylthioninium rescues cell death ((EC50 = 23 nM; *n* = 3) One-way ANOVA, Methylthioninium treatment: *P* < 0.0001, F = 109.7, DF = 4). (TIF 1286 kb)

Additional file 2: Supplementary Methods [76, 77]. (DOCX 17 kb)

Additional file 3: **Figure S2.** Reduction in Ca^2+^ influx through VGCCs reduces ATRA induced toxicity. **a** The effect of isradipine (VGCC inhibitor) in the toxicity assay (EC_50_ = 971 nM; *n* = 2). **b** The effect of U0126 (ERK1/2 kinase kinase (MEK) inhibitor) in the toxicity assay ((EC_50_ = 55.88 nM; *n* = 4) One-way ANOVA, U0126 treatment: *P* < 0.0001, F = 95.21, DF = 5). (TIF 110 kb)

Additional file 4: **Figure S3.** REM rescues ADDL induced cytotoxicity in primary hippocampal neurons. Viability assessment using live (yellow)/dead (purple) assay (representative pictures below the graphs – i, Control; ii, vehicle; iii, REM) of rat primary hippocampal neurons (Additional file 2: Supplementary Methods) exposed for 24 h to 1 μM ADDL’s treated with vehicle or 0.25 μM REM0043039 ((*n* = 3) Control/Vehicle: *p* = 0.0175, *t* = 3.9, DF = 4; Vehicle/REM: *P* = 0.0208, *t* = 3.704, DF = 4). (TIF 2806 kb)

Additional file 5: **Figure S4.** The farnesyl group of Rheb fits into Pde6δ’s hydrophobic pocket. **a** Rheb’s farnesyl moiety sequestered in Pde6δ internal hydrophobic cavity as solved in X-ray crystal structure. Pde6δ shown in ribbon (left) or solid surface (right), and farnesyl moiety fused to terminal cysteine shown in stick bonds. **b** Superimposition of the farnesyl moiety (purple) and farnesyl group as solved in the crystal structure (grey). (TIF 1245 kb)

Additional file 6: **Figure S5.** REM increases somatic and dendritic action potential AHP in hAPP brain slices. **a** K^+^ current analysis: left graph shows the total K^+^ current; right graph shows the 4-AP (I_A_ channel blocker) insensitive current (*n* = 7 mice per condition; RM-two way ANOVA 4-AP insensitive current amplitude: Interaction: *P* = 0.0294; F(15,180) = 1.865; DF = 15; Sidak’s multiple comparison test: 60 mV: *P* = 0.0496; *t* = 2.988; DF = 192; 70 mV: *P* = 0.0088; *t* = 3.512; DF = 192). **b** Dendritic single AP parameters in hAPP brain slices after at least one hour vehicle or REM incubation; example traces are shown in the insets next to the graphs. (*n* = 8 mice per condition; AHP amplitude: *P* = 0.0003; *t* = 4.816; DF = 14; AP decay slope: *P* = 0.0317; *t* = 2.387; DF = 14). (TIF 263 kb)

Additional file 7: **Figure S6.** REM decreases KCl depolarisation induced Ca^2+^ influx by modulation of L-type channels**.** Summary of Fura-2 fluorescence traces (ratio 340/380 nm over time) after normalising to the mean of the first minute “baseline” recording. (+ nifedipine: n = 8; − nifedipine *n* = 16). (TIF 197 kb)

Additional file 8: **Figure S7.** REM reduces behaviour deficits and phosphorylated Tau in R406W mice. **a** Western blot analysis showing the ratio of phosphorylated Tau on epitope AT8 (pS202, pT205) to total Tau in cortex of R406W transgenic mice treated with either vehicle or REM subcutanously for 4 weeks. Examples of the immunoblots are shown above the graph. (n = 8 or 9 vehicle or REM treated respectively; P = 0,0328; t = 2,324; DF = 17). **b** Average velocity of R406W Tau transgenic mice during a MWM test after 4 weeks treatment with REM. (*n* = 16 or 18 or 17 WT + Vehicle or R406W + Vehicle or R406W + REM respectively; WT + Vehicle/R406W + Vehicle: P = 0,0033; t = 3,181; DF = 32; R406W + REM/+Vehicle: *P* = 0,0307; t = 2,258; DF = 33). (TIF 160 kb)

Additional file 9: **Figure S8.** REM reaches effective concentrations in brains of hAPP mice and improves their ability to learn during a MWM test. **a** Search path length during the 4 training days in a MWM setting. (*n* = 12 mice per condition; multiple t-test: WT/hAPP Vehicle day 4: *P* = 0.033; *t* = 2.277; DF = 22; hAPP Vehicle/REM day 4: *P* = 0.05; *t* = 2.072; DF = 22). **b** Brain exposure levels of REM in hAPP mice 3 h post last dose. (TIF 142 kb)

## Abbreviations

ACI: Annulus crossing index
aCSF: Artificial cerebrospinal fluid
AD: Alzheimer’s disease
AHP: Afterhyperpolarisation
AP: Action potential
ATRA: All-trans retinoic acid
AUC: Area under the curve
Aβ: Amyloid-beta
Aβo: Soluble oligomeric Aβ
BSA: Bovine serum albumin
Ca^2+^: Calcium
DHPG: (S)-3,4-dihydroxyphenylglycine
DIC: Differential interference contrast
DIV: Days in-vitro
DMSO: Dimethyl sulfoxide
E: Embryonic day
ERK1/2: Extracellular signal-regulated kinase
FCS: Foetal calf serum
fEPSPs: Field excitatory postsynaptic potentials
GABA_B_: Gamma-aminobutyric acid receptor B
hAPP: Human mutant transgenic APP-V717I
hAPP*PS1: Human mutant transgenic APP-V717I*PS1-A246E
I_A_: Fast transient outward K^+^ current
K^+^: Potassium
KCl: Potassium chloride
LDH: Lactate dehydrogenase
LTD: Long-term depression
MAPK: Mitogen-activated protein kinase
ms: Millisecond
MWM: Morris water maze
NB: Neurobasal medium
PDL: Poly-D-lysine
REM-MTX: Methotrexate fused REM compound
RT: Room temperature
sc: Subcutaneous
TBS: Tris-buffered saline
TTX: Tetrodotoxin
WT: Wild-type
