# Supplementary figures and images for: Modifying Rap1-signalling by targeting Pde6δ is neuroprotective in models of Alzheimer’s disease
Source: Mol Neurodegener. 2018 Sep 26;13:50. doi: 10.1186/s13024-018-0283-3 (PMC6158915; doi:10.1186/s13024-018-0283-3)

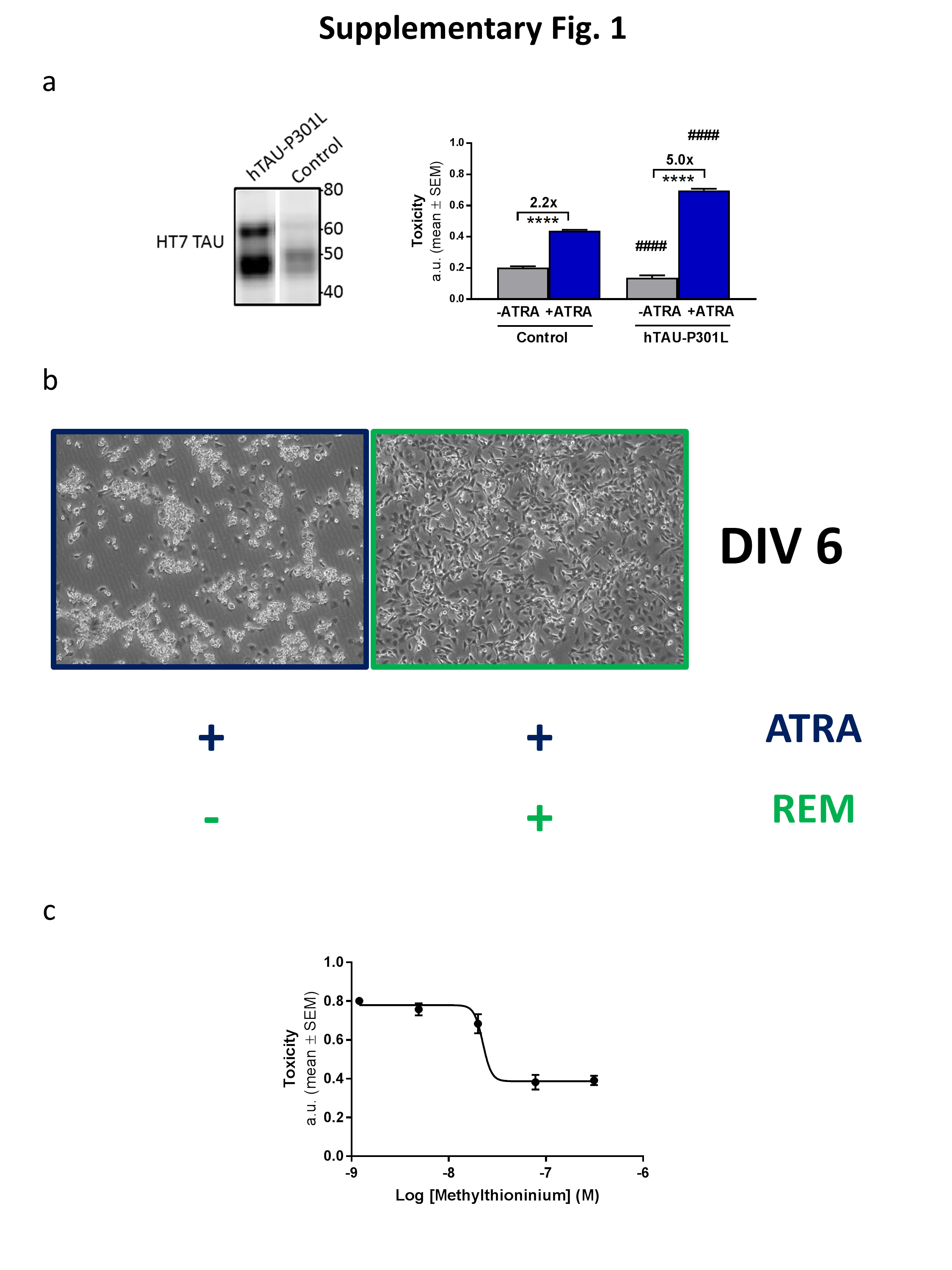

Supplement: Supplementary file 1 — Figure S1. ATRA induced cell death in the model is enhanced by expression of a cDNA encoding mutant human Tau and is prevented by REM. a Left: Western blot analysis confirmed successful tau overexpression (approximately 2-fold) in neuroblastoma cells containing the hTAU-P301L plasmid. Right: Toxicity was determined by quantifying LDH release in the medium in BE (2)-M17 neuroblastoma cells with (hTAU-P301L) and without (Control) expression of a cDNA encoding hTAUP301L incubated for 7 days with or without ATRA. The ATRA induced toxicity in hTAU-P301L cells represents the “toxicity assay” used in all following experiments (two-way ANOVA: +/− ATRA: P < 0.0001, F(1, 36)=4276, DF = 1; +/− hTAU-P301L: P < 0.0001, F (1, 36)=255.3, DF = 1; Sidak’s multiple comparison test: control +/− ATRA: P < 0.0001, t = 27.54, DF = 36; hTAU-P301L +/− ATRA: P < 0.0001, t = 64.93, DF = 36; n = 10). # denotes the effect of hTAU-P301L expression silencing compared to control cells, * indicates the effect of ATRA treatment. b Representative images of neuroblastoma cells exposed to ATRA for six days treated with either REM or vehicle. c Treating hTAU-P301L cells in the toxicity assay with tau aggregation inhibitior methylthioninium rescues cell death ((EC50 = 23 nM; n = 3) One-way ANOVA, Methylthioninium treatment: P < 0.0001, F = 109.7, DF = 4). (TIF 1286 kb) [file 13024_2018_283_MOESM1_ESM.tif]

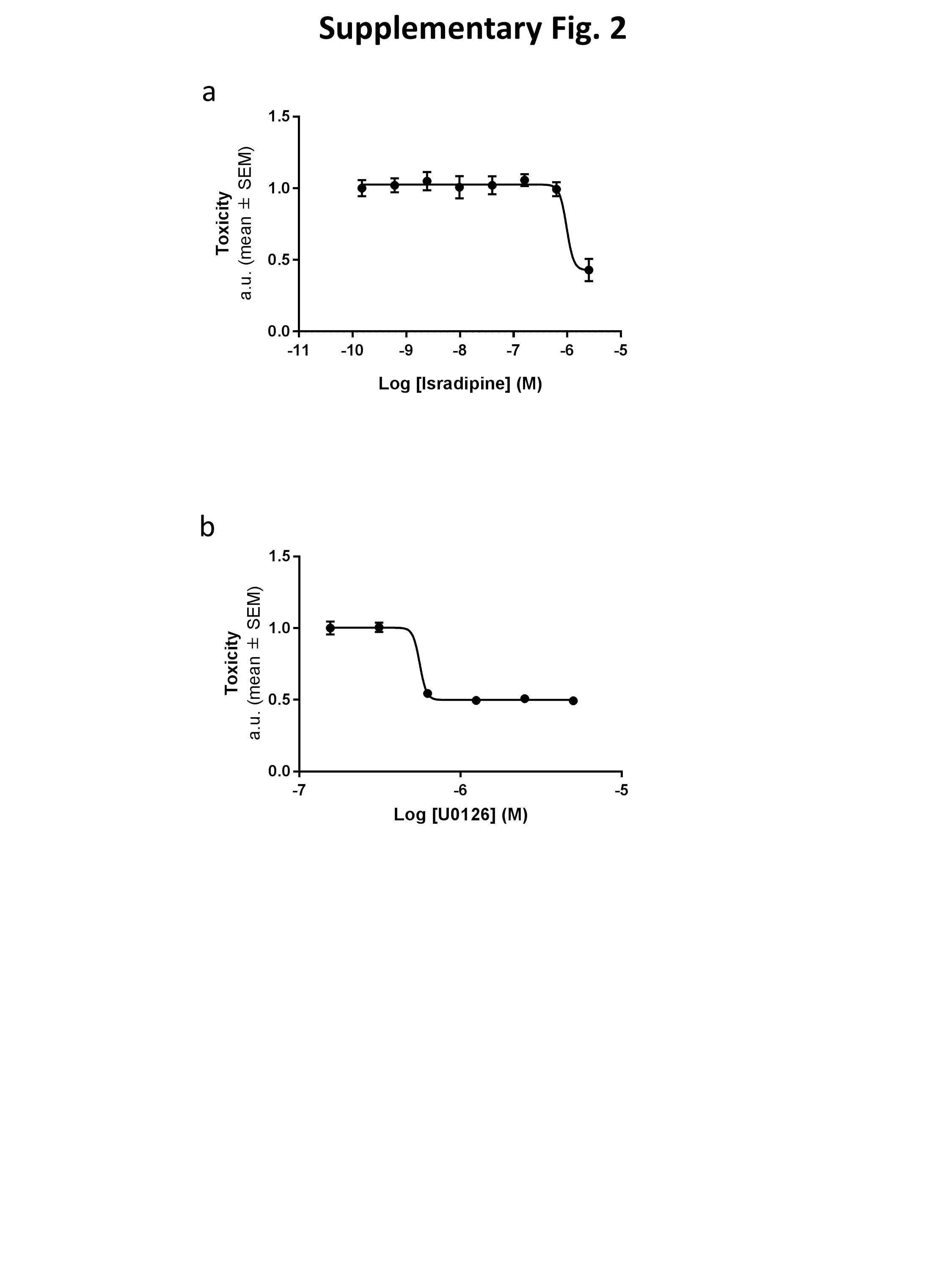

Supplement: Supplementary file 3 — Figure S2. Reduction in Ca2+ influx through VGCCs reduces ATRA induced toxicity. a The effect of isradipine (VGCC inhibitor) in the toxicity assay (EC50 = 971 nM; n = 2). b The effect of U0126 (ERK1/2 kinase kinase (MEK) inhibitor) in the toxicity assay ((EC50 = 55.88 nM; n = 4) One-way ANOVA, U0126 treatment: P < 0.0001, F = 95.21, DF = 5). (TIF 110 kb) [file 13024_2018_283_MOESM3_ESM.tif]

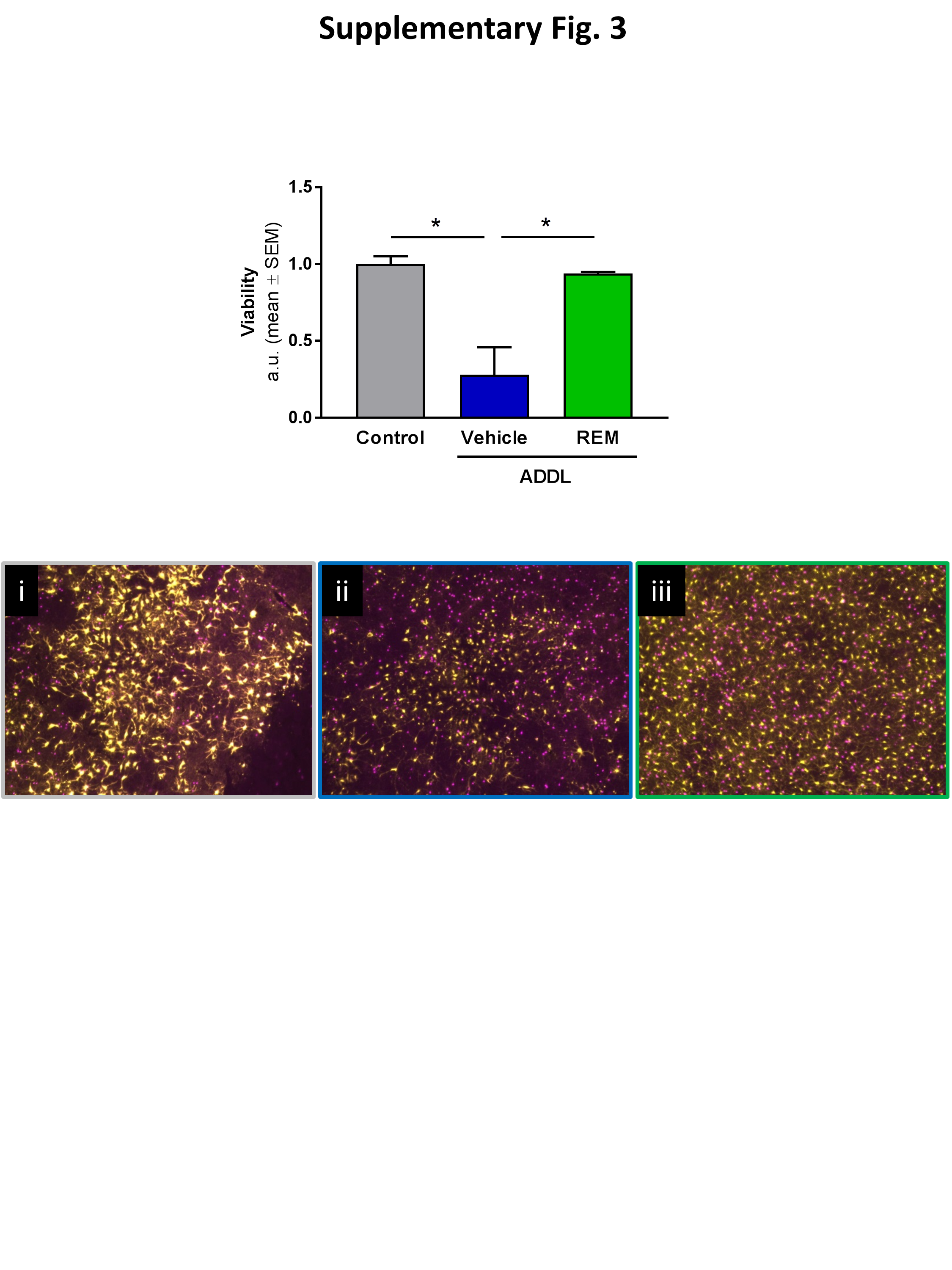

Supplement: Supplementary file 4 — Figure S3. REM rescues ADDL induced cytotoxicity in primary hippocampal neurons. Viability assessment using live (yellow)/dead (purple) assay (representative pictures below the graphs – i, Control; ii, vehicle; iii, REM) of rat primary hippocampal neurons (Additional file 2: Supplementary Methods) exposed for 24 h to 1 μM ADDL’s treated with vehicle or 0.25 μM REM0043039 ((n = 3) Control/Vehicle: p = 0.0175, t = 3.9, DF = 4; Vehicle/REM: P = 0.0208, t = 3.704, DF = 4). (TIF 2806 kb) [file 13024_2018_283_MOESM4_ESM.tif]

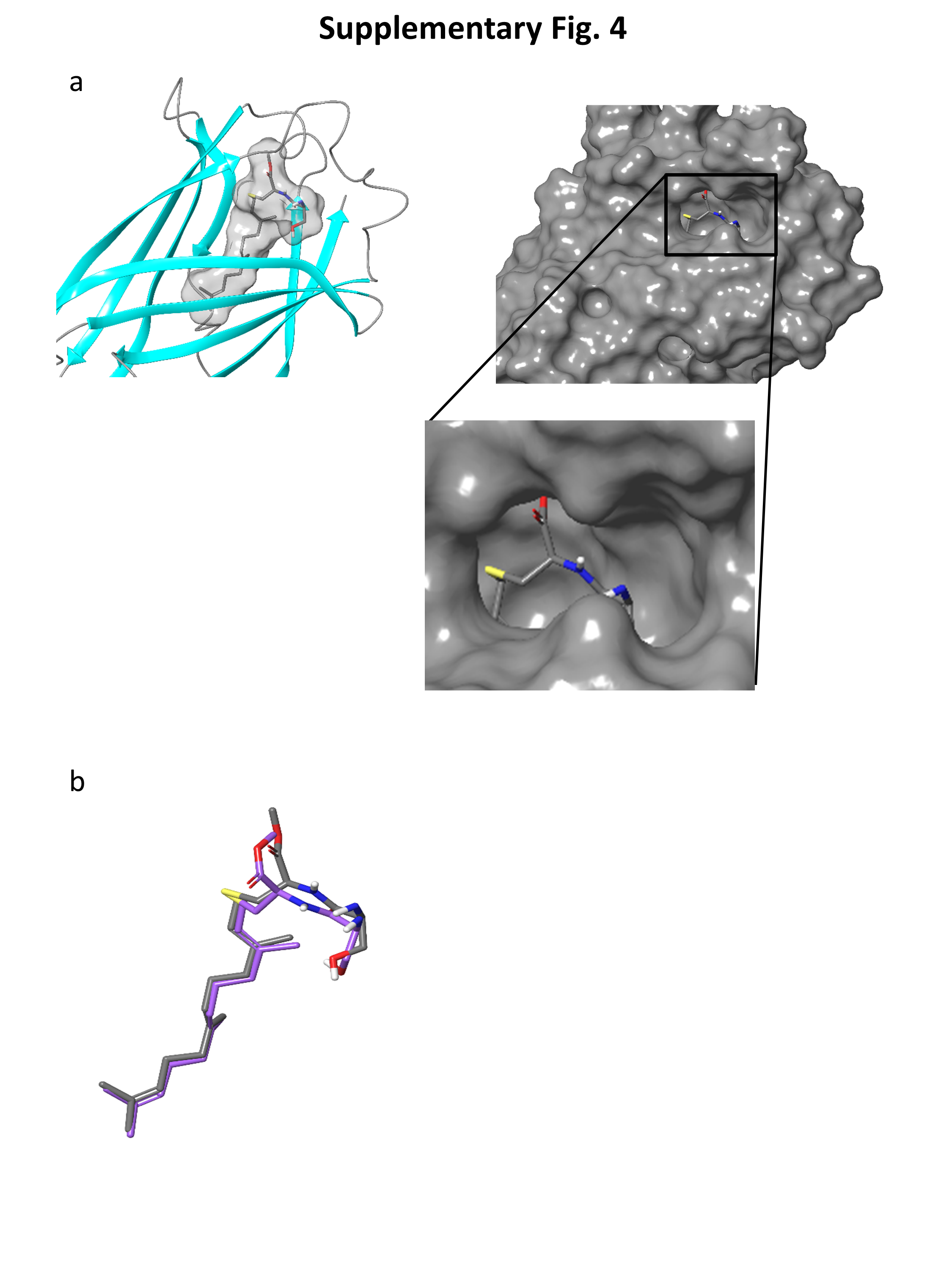

Supplement: Supplementary file 5 — Figure S4. The farnesyl group of Rheb fits into Pde6δ’s hydrophobic pocket. a Rheb’s farnesyl moiety sequestered in Pde6δ internal hydrophobic cavity as solved in X-ray crystal structure. Pde6δ shown in ribbon (left) or solid surface (right), and farnesyl moiety fused to terminal cysteine shown in stick bonds. b Superimposition of the farnesyl moiety (purple) and farnesyl group as solved in the crystal structure (grey). (TIF 1245 kb) [file 13024_2018_283_MOESM5_ESM.tif]

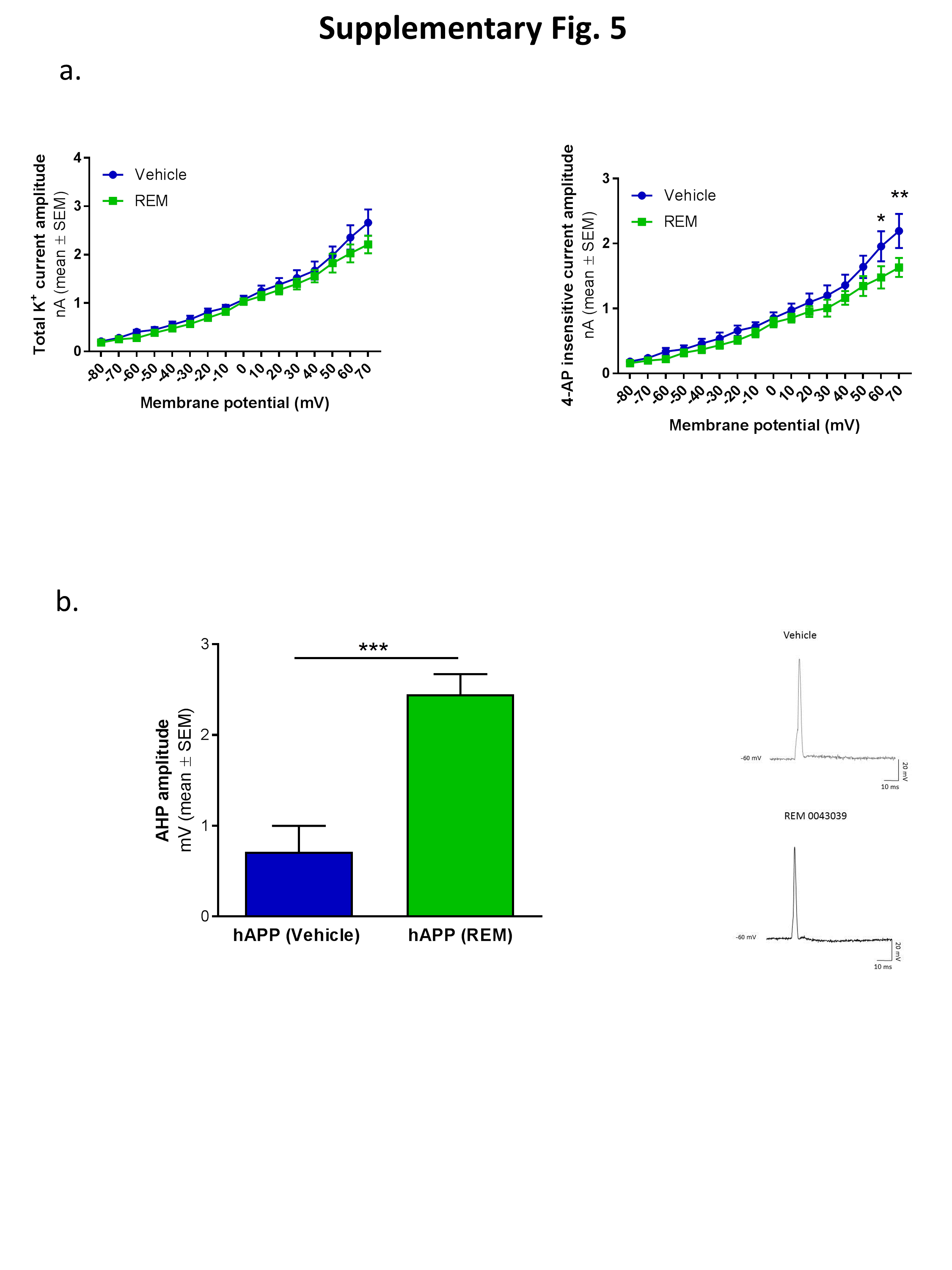

Supplement: Supplementary file 6 — Figure S5. REM increases somatic and dendritic action potential AHP in hAPP brain slices. a K+ current analysis: left graph shows the total K+ current; right graph shows the 4-AP (IA channel blocker) insensitive current (n = 7 mice per condition; RM-two way ANOVA 4-AP insensitive current amplitude: Interaction: P = 0.0294; F(15,180) = 1.865; DF = 15; Sidak’s multiple comparison test: 60 mV: P = 0.0496; t = 2.988; DF = 192; 70 mV: P = 0.0088; t = 3.512; DF = 192). b Dendritic single AP parameters in hAPP brain slices after at least one hour vehicle or REM incubation; example traces are shown in the insets next to the graphs. (n = 8 mice per condition; AHP amplitude: P = 0.0003; t = 4.816; DF = 14; AP decay slope: P = 0.0317; t = 2.387; DF = 14). (TIF 263 kb) [file 13024_2018_283_MOESM6_ESM.tif]

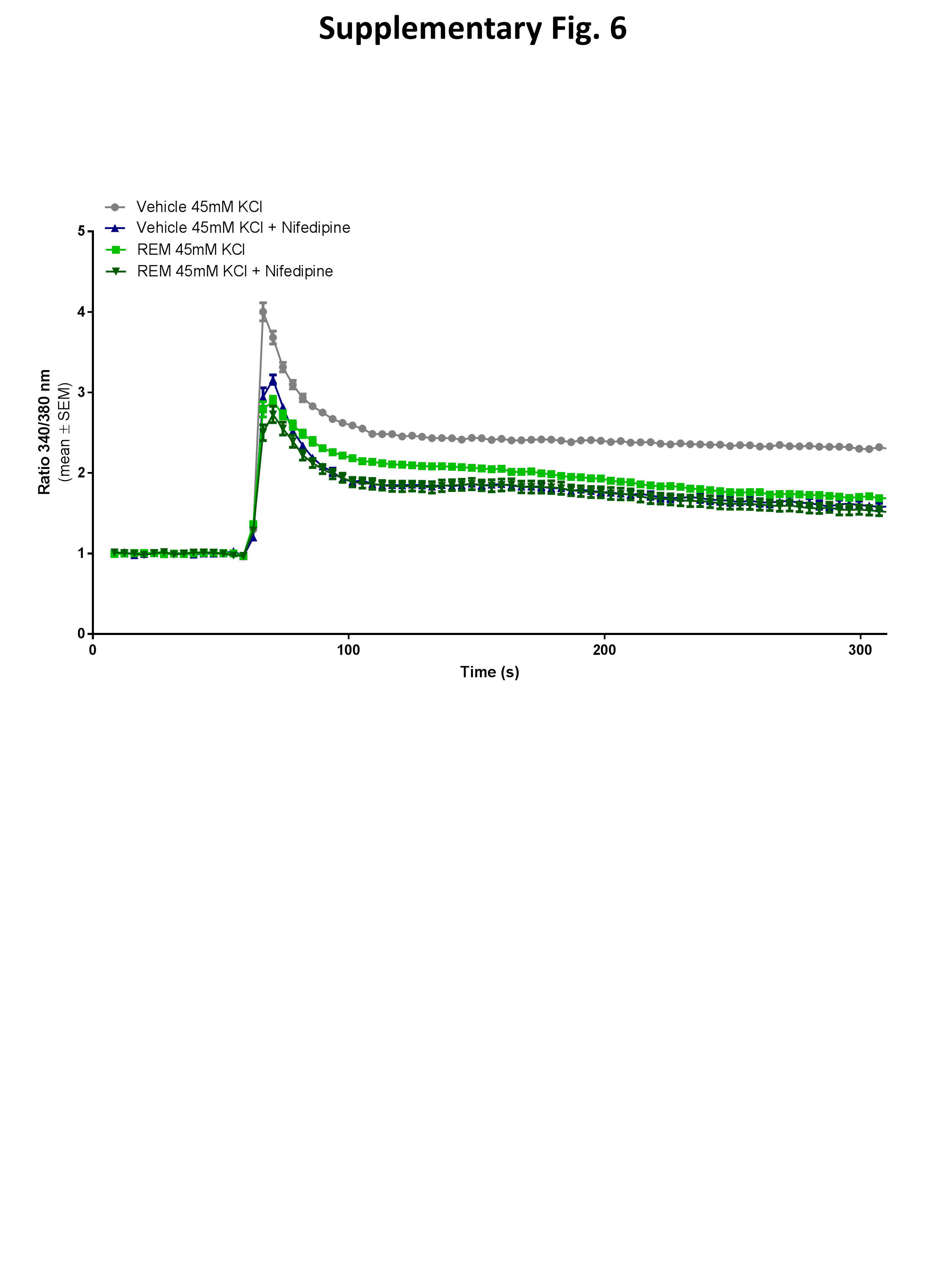

Supplement: Supplementary file 7 — Figure S6. REM decreases KCl depolarisation induced Ca2+ influx by modulation of L-type channels. Summary of Fura-2 fluorescence traces (ratio 340/380 nm over time) after normalising to the mean of the first minute “baseline” recording. (+ nifedipine: n = 8; − nifedipine n = 16). (TIF 197 kb) [file 13024_2018_283_MOESM7_ESM.tif]

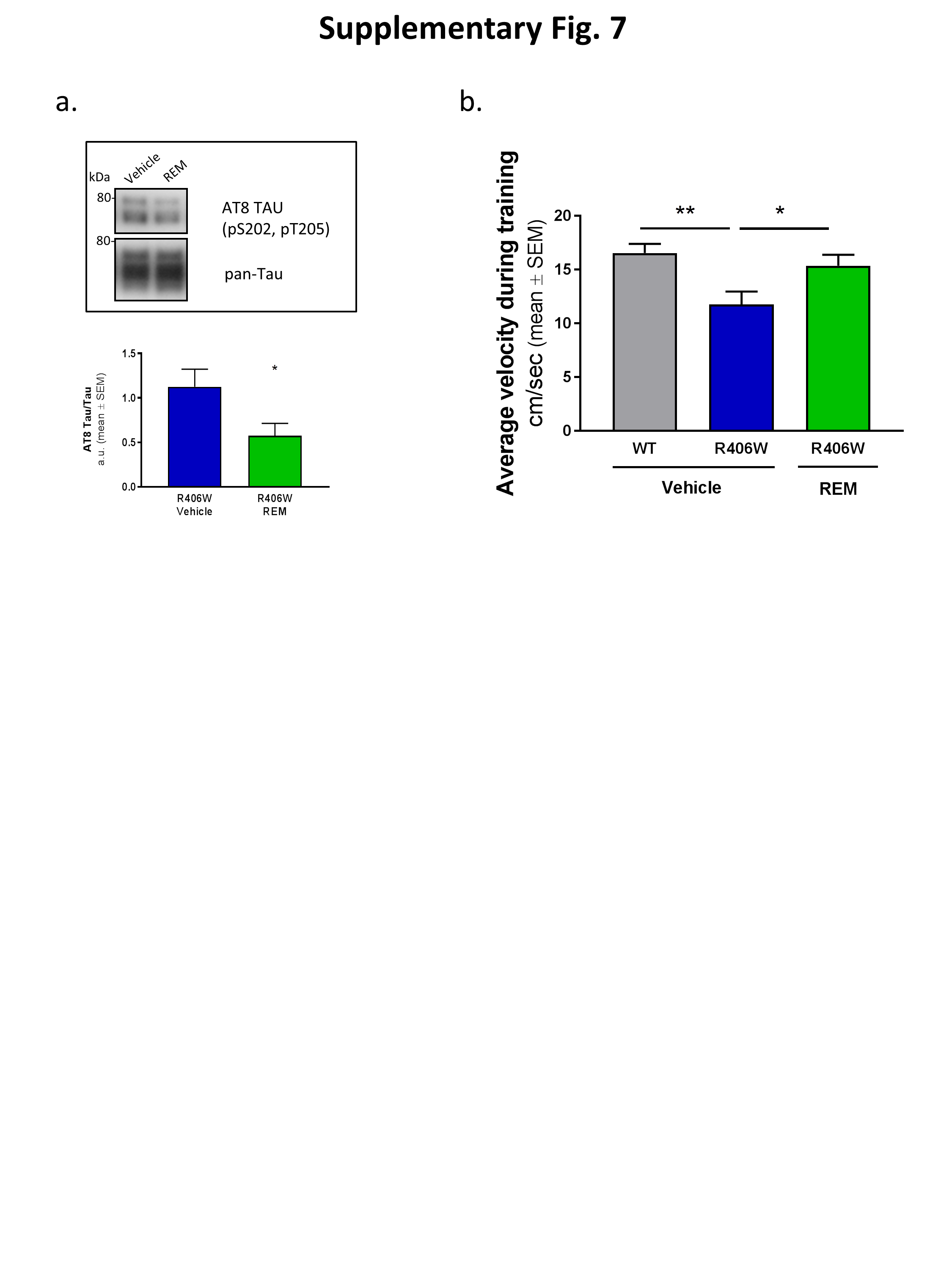

Supplement: Supplementary file 8 — Figure S7. REM reduces behaviour deficits and phosphorylated Tau in R406W mice. a Western blot analysis showing the ratio of phosphorylated Tau on epitope AT8 (pS202, pT205) to total Tau in cortex of R406W transgenic mice treated with either vehicle or REM subcutanously for 4 weeks. Examples of the immunoblots are shown above the graph. (n = 8 or 9 vehicle or REM treated respectively; P = 0,0328; t = 2,324; DF = 17). b Average velocity of R406W Tau transgenic mice during a MWM test after 4 weeks treatment with REM. (n = 16 or 18 or 17 WT + Vehicle or R406W + Vehicle or R406W + REM respectively; WT + Vehicle/R406W + Vehicle: P = 0,0033; t = 3,181; DF = 32; R406W + REM/+Vehicle: P = 0,0307; t = 2,258; DF = 33). (TIF 160 kb) [file 13024_2018_283_MOESM8_ESM.tif]

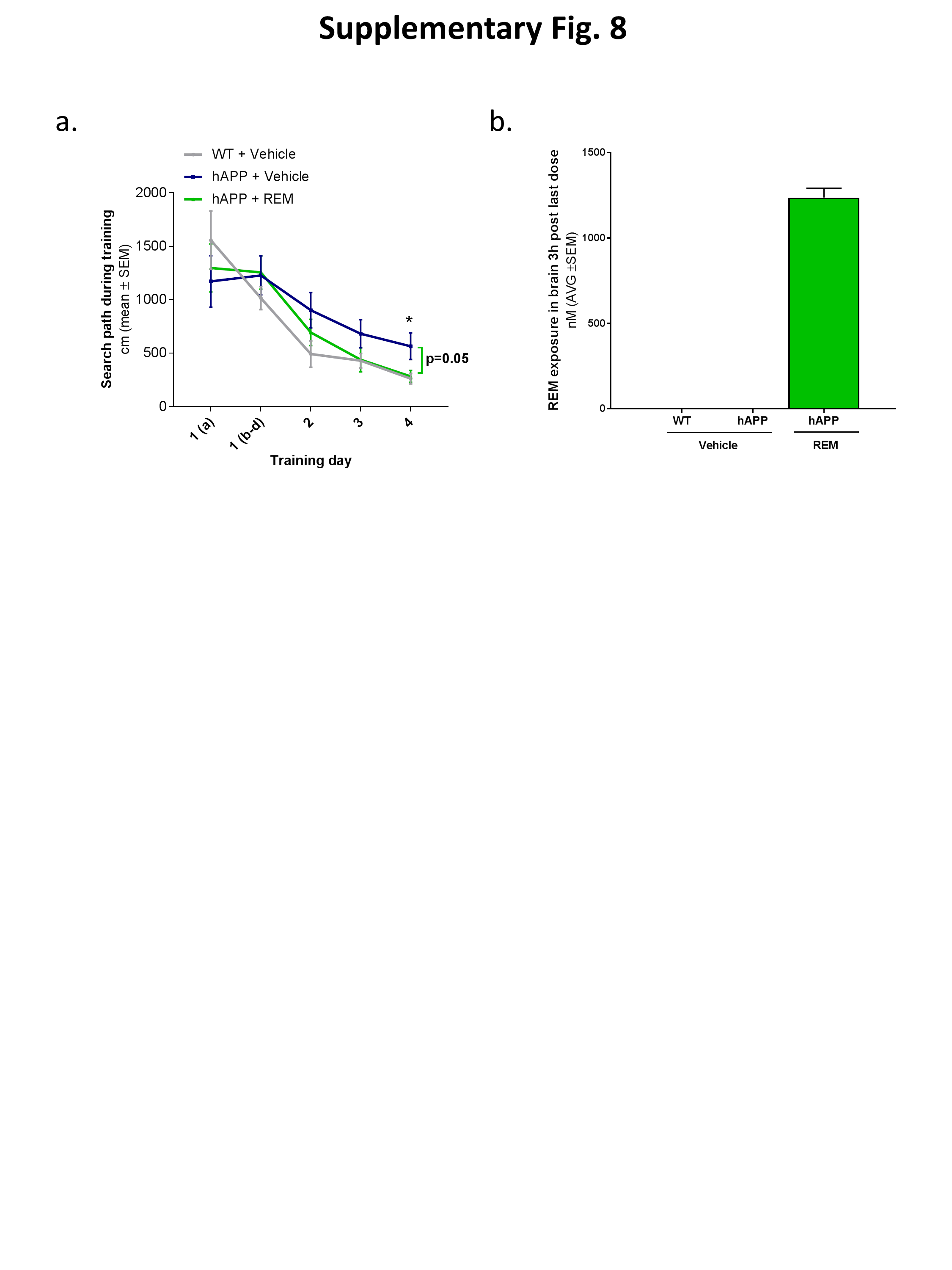

Supplement: Supplementary file 9 — Figure S8. REM reaches effective concentrations in brains of hAPP mice and improves their ability to learn during a MWM test. a Search path length during the 4 training days in a MWM setting. (n = 12 mice per condition; multiple t-test: WT/hAPP Vehicle day 4: P = 0.033; t = 2.277; DF = 22; hAPP Vehicle/REM day 4: P = 0.05; t = 2.072; DF = 22). b Brain exposure levels of REM in hAPP mice 3 h post last dose. (TIF 142 kb) [file 13024_2018_283_MOESM9_ESM.tif]
