# Supplementary material for: Modifying Rap1-signalling by targeting Pde6δ is neuroprotective in models of Alzheimer’s disease
Source: Mol Neurodegener. 2018 Sep 26;13:50. doi: 10.1186/s13024-018-0283-3 (PMC6158915; doi:10.1186/s13024-018-0283-3)
Supplement: Supplementary file 2 — Supplementary Methods [76, 77]. (DOCX 17 kb) [file 13024_2018_283_MOESM2_ESM.docx]

Additional file 9

Supplementary Methods:

**Aβo toxicity assay:**

DIV 19 rat primary hippocampal neurons were prepared as previously described^76^. Amyloid derived diffusible ligand (ADDL) preparation was done according to a protocol previously described by Klein^77^.

In short, Aβ 1-42 (AnaSpec Inc.) was dissolved in hexafluoroisopropanol (HFIP) to homogenise the peptide and HFIP was then evaporated completely during 10 minutes in a speedvac. The remaining Aβo film was dried overnight at -20°C over desiccant. Aβo film was then resolubilised in 100% DMSO anhydrous and further diluted (1/25) in Ham’s F12 medium (Caisson Labs). Blanks were prepared by adding equal amounts of DMSO to Ham’s F12 medium. Aβo and Blank solutions were incubated overnight at 4°C. The solution was then centrifuged at 14.000 x g for 10 minutes at 4°C and supernatant was transferred to fresh tubes and protein concentration determined by NanoDrop®. On DIV 21, primary rat hippocampal neurons were treated with equal volume of Blank/ADDL solution amounting to 0 and 1000 nM ADDL. Cultures were incubated at 37°C 5% CO_2_ for 24 hours before viability determination. Viability was assessed using the Live-Dead assay (Thermo Fisher) according to the manufacturer’s instructions.

**Synthesis procedure REM compounds**

5-(2,5-Difluorobenzyl)-N-(2-(5-fluoro-1H-indol-3-yl)ethyl)isoxazole-3-carboxamide (REM0043039)

N-Ethyldiisopropylamine (25.65 ml; 148.37 mmol) was added to a stirred mixture of 2-(5-fluoro-1H-indol-3-yl)ethan-1-amine hydrochloride (13.00 g; 59.35 mmol), 5-(2,5-difluorobenzyl)isoxazole-3-carboxylic acid (14.19 g; 59.35 mmol), and O-(7-azabenzotriazol-1-yl)-N,N,N',N'-tetramethyluronium hexafluorophosphate (22.57 g; 59.35 mmol) in dry dimethylfluoride (DMF; 90 ml). The mixture was stirred at room temperature for 60 hours and then was concentrated under reduced pressure. The residue was dissolved in ethyl acetate and the solution was washed with water and brine, dried over magnesium sulphate, filtered, and concentrated under reduced pressure. The residue was purified by flash chromatography on silica gel using a gradient of ethyl acetate (1-10%) in dichloromethane to give 20.02 g of a yellowish solid which was recrystallised in a mixture of dichloromethane and n-heptane to yield 19.06 g (80%) of 5-(2,5-difluorobenzyl)-N-(2-(5-fluoro-1H-indol-3-yl)ethyl)isoxazole-3-carboxamide as a white solid. ^1^H NMR (DMSO-d_6_) 10.92 (br. s., 1H), 8.82 (t, 1H), 7.1 -7.38 (m, 6H), 6.90 (td, 1H), 6.53 (s, 1H), 4.26 (s, 2H), 3.48 (q, 2H), 2.88 (t, 2H). ESI/APCI (+): 400 (M+H). ESI/APCI (-): 398 (M-H).

N-(2-(5-Chloro-1H-indol-3-yl) ethyl)-5-(2,5-difluorobenzyl)isoxazole-3-carboxamide (REM0042826)

Thionyl chloride (0.508 ml; 6.98 mmol) was added to the suspension of (2, 5-difluorobenzyl) isoxazole-3-carboxylic acid (0.200 g; 0.838 mmol) in chloroform (12 ml). The mixture was heated overnight at 80 °C and the solution was evaporated under reduced pressure. 2-(5-Chloro-1H-indol-3-yl) ethanamine hydrochloride (0.183 g; 0.776 mmol) followed by a solution of triethylamine (0.480 ml; 3.41 mmol) in dichloromethane (8 ml) were added to the resulting residue. The mixture was stirred at room temperature for 30 minutes and was washed with a solution of sodium carbonate. The organic layer was concentrated under reduced pressure and the residue was purified by flash column chromatography on silica gel (eluent: 2 to 10% of ethyl acetate in dichloromethane) to give 0.201 g (62%) of N-(2-(5-chloro-1H-indol-3-yl) ethyl)-5-(2,5-difluorobenzyl)isoxazole-3-carboxamide as a white solid. ESI/APCI (+):416 (M+H). ESI/APCI (-):415 (M-H). ^1^H NMR (DMSO-d_6_) 11.04 (1H, s); 8.82 (1H, t), 7.61 (1H, d), 7.33 (1H, d), 7.31(2H, m), 7.24 (1H, d), 7.22 (1H, m), 7.04 (1H, dd), 6.52 (1H, s), 4.25 (2H, s), 3.57(2H, m), 2.88 (2H, t).

A
